# Cloning and expression of selected ABC transporters from the *Arabidopsis thaliana* ABCG family in *Pichia pastoris*

**DOI:** 10.1371/journal.pone.0211156

**Published:** 2019-01-18

**Authors:** Katharina Gräfe, Kalpana Shanmugarajah, Thomas Zobel, Stefanie Weidtkamp-Peters, Diana Kleinschrodt, Sander H. J. Smits, Lutz Schmitt

**Affiliations:** 1 Institute of Biochemistry, Heinrich Heine University, Düsseldorf, Germany; 2 Cluster of Excellence on Plant Sciences, Heinrich Heine University, Düsseldorf, Germany; 3 Center for Advanced Imaging, Heinrich Heine University, Duüsseldorf, Germany; 4 Protein Production Facility, Heinrich Heine University, Duüsseldorf, Germany; University of Cambridge, UNITED KINGDOM

## Abstract

Phytohormones play a major role in plant growth and development. They are in most cases not synthesized in their target location and hence need to be transported to the site of action, by for instance ATP-binding cassette transporters. Within the ATP-binding cassette transporter family, Pleiotropic Drug Resistance transporters are known to be involved in phytohormone transport. Interestingly, PDRs are only present in plants and fungi. In contrast to fungi, there are few biochemical studies of plant PDRs and one major reason is that suitable overexpression systems have not been identified. In this study, we evaluate the expression system *Pichia pastoris* for heterologous overexpression of *PDR* genes of the model plant *Arabidopsis thaliana*. We successfully cloned and expressed the potential phytohormone transporters PDR2 and PDR8 in *P*. *pastoris*. Sucrose gradient centrifugation confirmed that the overexpressed proteins were correctly targeted to the plasma membrane of *P*. *pastoris* and initial functional studies demonstrated ATPase activity for WBC1. However, difficulties in cloning and heterologous overexpression might be particular obstacles of the PDR family, since cloning and overexpression of *White Brown Complex 1*, a half-size transporter of the same ABCG subfamily with comparable domain organization, was more easily achieved. We present strategies and highlight critical factors to successfully clone plant *PDR* genes and heterologously expressed in *P*. *pastoris*.

## Introduction

Phytohormones (plant hormones) are signal molecules which enable plants to adapt growth and development to internal and external stimuli [1]. They comprise various molecule classes like abscisic acid (ABA), auxin, cytokinin, gibberellin, jasmonic acid, ethylene, strigolactones or salicylic acid [2]. In most cases the site of action is distant from the site of production, which requires transport across membranes. Many ATP-binding cassette (ABC) transporters, have been shown to be involved in hormone transport in plants [3–5].

ABC transporters are ubiquitous in all kingdoms of life and facilitate the transport of a large variety of substrates across membranes using ATP hydrolysis as energy source [6, 7]. They are composed of four core domains: two nucleotide-binding domains (NBDs) and two transmembrane domains (TMDs). NBDs comprise all the sequence motifs required for ATP hydrolysis, while the TMDs are composed of 4 to 10 α-helices and form the substrate translocation pathway across the lipid bilayer. These four domains can be either encoded on a single gene (full-size transporter), or one NBD and one TMD are fused on one gene (half-size transporter), or all the domains are encoded by separate genes [8–12].

Compared to other organism, the ABC protein family is significantly enlarged in plants with about 130 members in *Arabidopsis thaliana* and *Oryza sativa* [11, 13, 14]. The ABC proteins are classified into eight subfamilies (ABCA–ABCG, ABCI) with ABCG subfamily being the largest [15] and showing a reverse domain orientation (NBD N-terminal to the TMD) compared to the other subfamilies. In Arabidopsis, the ABCG subfamily comprises 28 half-size transporters (White Brown Complex (WBC)) and 15 plant- and fungi-specific Pleiotropic Drug Resistance (PDR) full-size transporters [11]. Potential roles include heavy metal detoxification [16, 17], pathogen response [18–23] and formation of physical barriers [24–26].

Interestingly, members of the ABCG-subfamily and especially the full-size transporters were also shown to be involved in phytohormone transport [27–31]. The phytohormone ABA is involved in environmental stress response and plant development [32]. In Arabidopsis, the ABCG full-size transporters AtPDR2, AtPDR3, AtPDR12 and the half-size transporter AtWBC25 were reported to mediate ABA transport from the endosperm to the seeds [33]. It was postulated that AtPDR2 and AtPDR12 function as importers [30, 33], which is unusual for eukaryotic ABC transporters. Cytokinin and auxin regulate the overal growth and development in plants [2]. AtWBC14 was shown to be essential for cytokinin root-to-shoot translocation [34, 35], while AtPDR8 and AtPDR9 mediate the transport of the auxin precurser indole-3-butyric acid. It was hypothisyzed that the transporters function in hormon homeostasis [27, 29]. Another phytohormone potentially transported by a PDR is strigolactone. In *Petunia hybrida* PhPDR1 mediates the export of strigolactone, which regulates the axillary branching and the cultivation of arbuscular mycorrhizae [31]. However, identification of transported substrates by reverse genetics and knock-out analysis in plants is complicated since most *PDR* mutants exhibit pleiotropic phenotypes. For instance, AtPDR8 confers, besides a potential role in indole-3-butyric acid transport, resistance to heavy metals as well as salt and drought stress [16, 36]. In addition to that, Lu *et al*. reported that AtPDR8 transports a product of the PENETRATION 2 myrosinase pathway and contributes thereby to pathogen defense [37]. Likewise, AtPDR2 was, apart from ABA transport, reported to be involved in root exudation of secondary metabolites and thereby shape the rhizosphere microbiome [38].

Based on these studies, it is often difficult to conclusively demonstrate whether a single or various substrates are transported. Furthermore, the new emerging role of ABC transporters in plants as importers requires clarification about the transport mechanisms. However, biochemical studies, helping to better understand the proteins and the transport process are rather limited. One reason is the necessity for purified protein in adequate quantities for biochemical and/or structural studies. Hence, it is imperative to have a suitable overexpression system that provides sufficient amounts of the protein in reasonable time. This is still a main issue concerning eukaryotic membrane proteins. Heterologous overexpression of membrane proteins is often challenging due to the size and hydrophobic nature of membrane proteins, toxicity of the protein to the host, misfolding, wrong localization or lack of the proper post-translational modification(s) [19, 39–41]. Other studies also demonstrated that not only expression, but also cloning of membrane proteins, especially ABC transporters, may cause difficulties because the sequences are unstable in *Escherichia coli* [19, 42–46]. Despite these challenges, heterologous overexpression is for some subsequent applications indispensable. Homologous expression has the advantage that the protein is in its native environment, including the lipid environment, proper post-translational modification(s) and correct subcellular localization. Plant expression systems, however, often exhibit slow growth and low expression yields of the desired protein, which is unfavorable for *in vitro* studies. Heterologous expression systems, like bacterial or yeast based systems, in contrast have the advantage of fast protein production with higher yields as well as established initial purification protocols [41]. For heterologous expression of eukaryotic membrane proteins eukaryotic expression systems are in many cases more suitable over bacterial systems regarding toxicity to the host cell, posttranslational modifications, codon usage and proper folding [39, 47–51]. Insect cell as well as plant cell based systems were successfully used for the expression of several plant membrane proteins [52–54], although their major drawbacks are time consumption and/or costs and sophisticated handling. Compared to that, yeast expression systems are a good alternative, they exhibit a eukaryotic protein processing machinery but are also fast growing and easy to handle [55]. The bakers yeast *Saccharomyces cerevisiae* is an often-used system for heterologous overexpression of eukaryotic membrane proteins [56–59]. Recently, various protein structures of membrane proteins were determined in which the yeast expression system *Pichia pastoris* (reclassified as *Komagataella pastoris*) was used for heterologous expression of membrane proteins [55], indicating its ability to produce correctly folded and processed proteins in substantial amounts. Furthermore, the same system was used to express 25 human ABC transporters [60]. Coherent major advantages are the ability (1) to grow to very high biomass concentrations, which are (2) accomplished in a reasonable time, (3) the possibility to use bioreactors, which allow control about the fermentation process by monitoring cultivation parameters, (4) the methylotrophic lifestyle, including the very strong and tightly regulated AOX1 promoter [61] and (5) its positive record in expression of affinity-tagged membrane proteins for subsequent purification and biochemical applications [60, 62, 63].

Members of the PDR family were expressed in eukaryotic systems [27, 33, 64], however, other studies also demonstrated that expression of PDRs is not always easily achieved. For example *At*ABCG37/PDR9 did not localize to the correct membranes when expressed in *S*. *cerevisiae*. It was subsequently expressed in HeLa cells and in *Schizosaccharomyces pombe* [27]. *Nicotiana plumbaginifolia* PDR5 could neither be expressed in *S*. *cerevisiae* nor in *S*. *pombe* [65]. Expression of *Nt*PDR1 in the heterologous system *S*. *cerevisiae* was weak and unstable and the protein was not correctly localized. It was finally expressed in the homologous system. For *N*. *plumbaginifolia* PDR1, even cloning was not successful [19].

Here, we demonstrate the successful heterologous overexpression of *At*ABCG30/PDR2, *At*ABCG36/PDR8 and the half-size transporter *At*ABCG1/WBC1 using the *P*. *pastoris* expression system. The accomplishment of cloning and expression of large plant membrane proteins with high numbers of transmembrane helices is not always easily achieved. As described above, problems often occur, but detailed reports how to realize cloning and expression are still rare. In this study, we evaluate the *P*. *pastoris* expression system for heterologous expression of plant ABC transporters belonging to the PDR subfamily and employed *P*. *pastoris* as a host for this protein family.

## Materials and methods

### Accession numbers and cDNA

Sequence data in this article can be found in The Arabidopsis Information Resource (TAIR) database under accession numbers AT4G15230 (*At*ABCG30/PDR2), AT2G26910 (*At*ABCG32/PDR4), AT2G37280 (*At*ABCG33/PDR5), AT2G36380 (*At*ABCG34/PDR6), AT1G15210 (*At*ABCG35/PDR7), AT1G59870 (*At*ABCG36/PDR8), and AT2G39350 (WBC1) [66]. The copy DNA (cDNA) of *A*. *thaliana* ecotype Columbia-0 was kindly provided by Prof. Andreas Weber, Institute for Plant Biochemistry and Prof. Peter Westhoff, Institute of Developmental and Molecular Biology of Plants, Heinrich Heine University Düsseldorf.

### Growth conditions and strains

*E*. *coli* strains were grown on LB medium (0.5% (w/v) yeast extract, 1% tryptone (w/v), 1% NaCl, 1.5% (w/v) agar for plates) supplemented with zeocin (25 μg/ml), kanamycin (50 μg/ml) or ampicillin (100 μg/ml), depending on the resistance gene encoded on the plasmid used for transformation, and incubated at 37°C. Liquid cultures were shaken at 180 rpm. The *E*. *coli* strains DH5α (Thermo Fisher Scientific), XL1-Blue, XL10-Gold, SURE 2 (all from Agilent Technologies) NEB Turbo (New England Biolabs, NEB) and CopyCutter EPI400 (epicentre) were used in this work. *P*. *pastoris* strain X33 (Invitrogen) and *S*. *cerevisiae* strain YPH500 [67] were grown on yeast peptone dextrose (YPD) medium (1% yeast extract (w/v), 2% tryptone (w/v), 2% glucose (w/v), 2% (w/v) agar for plates) at 30°C. *P*. *pastoris* liquid cultures were grown in minimal glycerol medium (1.34% yeast nitrogen base (w/v), 1% glycerol (w/v), 4 x 10^−5^% (w/v) biotin) at 30°C and 200 rpm. The buffered glycerol complex medium was composed of 100 mM potassium phosphate, 1% (w/v) yeast extract, 2% (w/v) peptone, 1.34% yeast nitrogen base, 4 x 10^−5^% (w/v) biotin, and 1% glycerol (v/v). For induction of protein expression, glycerol was substituted by 0.5% (v/v) methanol in minimal as well as in complex media.

### Primer design and polymerase chain reaction

The Clone Manager Suite (Sci-ED Software) was used for primer design. If required for cloning, overhangs were fused to the primers adding, for example, restriction sites to the sequence. All oligonucleotides were synthesized by MWG Eurofins. The polymerase chain reaction (PCR) was performed in a Biometra TGradient cycler using standard protocols. The optimal annealing temperatures were determined by temperature screening. All PCR products were extracted from agarose gels (Gel purification kit, Macherey-Nagel). DNA was sequenced by GATC Biotech.

### Cloning

The enzymes used for restriction digestion of the respective constructs were all obtained from NEB. T4 DNA ligase (NEB) was used for ligation of digested fragments. The reactions were set up according to the manufacturer’s protocols except that ligation was performed overnight at 4°C. In-Fusion cloning (Clonetech) was performed according to the manufacturer’s protocols with the modification that the reaction was incubated for 30 min and transformed bacteria was grown at 30°C. *E*. *coli* XL-10Gold or CopyCutter EPI400 competent cells were transformed by the respective cloning reaction using standard heat shock protocols. CopyCutter EPI400 cells were grown overnight, the induction solution (epicentre) was added and cells were grown for another 4–5 h. Constructs were isolated with a plasmid isolation kit (Macharey and Nagel) and confirmed by sequencing.

Homologous recombination in *E*. *coli* was performed according to Datsenko and Wanner (2000) [68]. Briefly, electro competent Bw25113 cells were transformed by the helper plasmid pKD46. Positive clones were selected on ampicillin-containing agar plates and were made electro competent again. These cells were transformed by the expression vector and by PCR fragments that contained the *PDR* coding sequence flanked by sequences that were homologous to the expression vector. Positive clones were selected on plates containing the respective antibiotic. For removing pKD46 plasmid, cells were incubated at 43°C. Success of transformation was confirmed by colony PCR. Homologous recombination *in S*. *cerevisiae* was performed similarly as described in Schiestl *et al*. (1989) [69]. Briefly, a 3 ml YPH500 overnight culture was harvested (5,000 x g, 1 min) and the following was added to the pellet: 10 μl carrier DNA (Clontech), 500 ng insert, 100 ng linearized expression vector, 500 μl LP-mix (40% polyethylene glycol 4000, 100 mM lithium acetate, 10 mM Tris-HCl pH 7.5 1 mM ethylenediaminetetraacetic acid (EDTA)), and 55 μl dimethyl sulfoxide. The mixture was incubated at 25°C for 30 min followed by a heat shock at 42°C for 15 min. 500 μl TE-buffer pH 7.5 were added and the cells were pelleted (5,000 x g, 1 min). The cells were washed with 1 ml TE-buffer (10,000 x g, 30 sec.), re-suspended in 500 μl YPD medium and regenerated for 1 h at 30°C, 180 rpm. The culture was plated on SC minus uracil medium and incubated at 30°C until colonies formed (2–3 days).

### Transformation of *P*. *pastoris*

*P*. *pastoris* was transformed by the expression vectors according to the guidelines of Invitrogen. Briefly, 20 μg of the respective plasmids were linearized with *Mss*I (Thermo Fisher Scientific) and used to transform 80 μl electro-competent *P*. *pastoris* cells. Clones were selected on YPD-agar supplemented with 500 μg/ml zeocin. Single clones were re-streaked on separate YPD plates.

### Colony PCR

One transformed *P*. *pastoris* colony was mixed with 25 μl water and 5 μl lysing enzyme solution (10 mg/ml lysing enzymes (Sigma Aldrich) in 100 mM Na_3_PO_4_ pH 7.4, 1 mM dithiothreitol) and incubated at 30°C for 30 min. The mix was flash-frozen in liquid nitrogen twice. For the PCR reaction, 3 μl of the mix were added to the PCR-mix. The PCR was performed using standard protocols and the products were analyzed by agarose gel electrophoresis.

### Expression screening of transformed *P*. *pastoris* clones

Expression screenings were performed as described in Ellinger *et al*. (2013) [44]. Minimal glycerol medium (50 ml) was inoculated with several colonies of the same clone and shaken at 220 rpm and 30°C for 24 h. Expression was induced by changing to minimal methanol medium. After another 24 h, 10 ml of cells were harvested by centrifugation (3,000 x g, 10 min, 4°C) and re-suspended in 4 ml ice-cold extraction buffer (50 mM Tris-HCl pH 7.5, 200 mM NaCl, 330 mM sucrose, 1 mM EDTA, 1 mM ethylene glycol-bis(β-aminoethyl ether)-N,N,N′,N′-tetraacetic acid). The cells were centrifuged again and re-suspended in 0.5 ml extraction buffer. Zirconia beads (Roth) equivalent to 1/3 of the total volume were added. Cells were disrupted by vortexing 6 times for 1 min and chilling on ice between the disruption cycles. Cell debris was removed by centrifugation (12,000 x g, 5 min, 4°C) and MgCl_2_ was added to the supernatant to a final concentration of 10 mM. After incubation for 15 min on ice, the membranes were pelleted by centrifugation (90 min at 20,000 x g, 4°C). The supernatant was removed and the membranes were re-suspended in extraction buffer. Expression was analyzed by SDS-PAGE and Western Blotting.

### Fermentation

Cells were fermented in a 15 l table-top glass fermenter (Applikon Biotechnology) according to the Invitrogen Pichia Fermentation Process Guidelines. Cells were grown overnight in 1 l minimal glycerol medium and used to inoculate 6 l basal salt medium. The fermentation was performed at 30°C and aeration was kept greater than 20% oxygen saturation. After a glycerol fed-batch of 50% glycerol for 5 h, protein expression was induced by addition of methanol (3.6 ml/h/l of culture). After 48 h, cells were harvested by centrifugation (5,000 x g, 10 min, 4°C), frozen in liquid nitrogen and stored at -80°C for subsequent use.

### Isolation of crude membrane vesicles from *P*. *pastoris*

*P*. *pastoris* wet cells expressing PDR8, PDR2, WBC1 or MDR3 were thawed on ice and re-suspended with extraction buffer (50 mM Tris-HCl pH 7.5, 200 mM NaCl, 330 mM sucrose, 1 mM EDTA, 1 mM ethylene glycol-bis(β-aminoethyl ether)-N,N,N′,N′-tetraacetic acid, supplemented with protease inhibitor cocktail (Roche)) to a final concentration of 0.5 g cells/ml. The cells were passed two times through a pre-cooled TS Series Cell Disruptor (Constant System) at 2.5 kbar. The disrupted cells were centrifuged twice (15,000 x g, 30 min, 4°C). Subsequently, the membranes were centrifuged for 1 h at 125,000 x g, 4°C. The membranes were re-suspended in buffer A (50 mM Tris-HCl pH 8, 150 mM NaCl, 15% glycerol), frozen in liquid nitrogen and stored at -80°C.

### Isolation of crude membranes from *S*. *cerevisiae*

*S*. *cerevisiae* cells were grown in YPD medium at 30°C. When the OD had reached 1.5 the nitrogen source was replenished by addition of 10% of 5 x YP (50 g/l yeast extract; 100 g/l tryptone/peptone). Cells were harvested at an OD of approximately 3.5. The isolation of crude membranes was adapted from [70, 71]. All steps were performed on ice or in cold rooms. Centrifuges were pre-cooled to 4°C. Briefly, 30 g wet cells were resuspended in extraction buffer supplemented with protease inhibitor cocktail (Roche). The cells were disrupted 5 times for 1 min in a bead mill homogenizer (BioSpec) and incubated on ice between the disruptions steps. Unbroken cells were removed by 3 centrifugation steps (1,000 x g, 2 x 5 min; 3,000 x g, 5 min). Subsequently, the supernatant was centrifuged for 1 h at 21,000 x g. The resulting pellet was re-suspended in 5 ml buffer A, frozen in liquid nitrogen and stored at -80°C.

### Subcellular fractionation of crude membranes

To determine the localization of PDR2, PDR8 and WBC1 in *P*. *pastoris*, crude membranes were diluted to 2 mg/ml in 1 ml ice-cold hypo-osmotic buffer (50 mM Tris-HCl pH 7.5, 200 mM sorbitol, 1 mM EDTA, protease inhibitor cocktail (Roche)) and layered on the top of a continuous sucrose gradient. MDR3 and PDR5 crude membranes were used as plasma membrane markers [44, 72]. The gradients were created using an ÄKTAprime plus pump system (GE Healthcare Life Sciences) generating a linear gradient ranging from 22 to 60% sucrose in 10 mM Tris-HCl, pH 7.5, 1 mM EDTA, 800 mM sorbitol. The different membrane species were separated by ultracentrifugation (130,000 x g, 22 h, 4°C) in a swing-out rotor. The gradients were split into 500 μl aliquots and analyzed by SDS-PAGE and Western Blotting.

### Cloning and expression of eGFP fusion proteins in *P*. *pastoris*

The Green Fluorescent Protein (eGFP) was fused to the N-terminus of PDR2, PDR8 and WBC1. Therefore, the e*GFP* coding sequence was cloned into the pSGP18 expression vector upstream of the respective genes. The e*GFP* gene was synthesized as a codon optimized version for *P*. *pastoris* and cloned into pSGP18-PDR2-Ntag and pSGP18-PDR8-Ntag by Genscript. In order to generate the construct for expression of free eGFP a stop codon was integrated downstream of the eGFP coding sequence by site directed mutagenesis PCR. All eGFP constructs were transformed into *P*. *pastoris* as described above. For expression, one fully-grown plate of the respective clone was used to inoculate 500 ml minimal glycerol medium in a 2 L baffled flask. After 24 h incubation the medium was exchanged to minimal methanol medium and thereby expression of the eGFP fusion proteins was induced. After 24 h, samples of the different cultures were taken and diluted to equal optical densities. Samples were immediately subjected to confocal fluorescence microscopy.

### Confocal fluorescence microscopy

*P*. *pastoris* cultures expressing the respective eGFP fusion protein were mixed with 0.1% (v/v) of the fluorescence stain SCRI Renaissance 2200 (SR2200) [73] and pipetted onto a poly-L-lysine coated slide (Thermo Scientific) and covered with a coverslip. Images were taken on a Zeiss LSM880 microscope with Airyscan and a 63 x NA 1.4 objective. For image acquisition the SR-Airyscan mode (pixel size of 40 nm) was used and the resulting images were automatically processed using the ZEN software. Figures were created using OERO.figure [74]. eGFP excitation was performed at 488 nm and SR2200 at 404 nm.

### ATPase activity measurement of WBC1

Heterologously expressed WBC1 and WBC1-EQ/HA was purified by calmodulin binding peptide (CBP) affinity chromatography as described elsewhere [44]. The yield was 2 mg per 100 g wet cells. Subsequently, ATPase activity of WBC1 was determined with the malachite green assay [75]. Reactions were performed in a total volume of 25 μl in reaction buffer containing 10 mM MgCl_2_ and 1 μg purified WBC1. The reaction was started after adding 5 mM ATP and stopped after 40 min at 25°C by transfer into 175 μl of 20 mM H_2_SO_4_. Afterwards, 50 μl dye solution was added to quantify the amount of free phosphate spectroscopically at 595 nm. A Na_2_HPO_4_ standard curve was used for the calibration of free phosphate.

### Polyacrylamide gel electrophoresis and Western Blotting

Protein expression was analyzed by SDS-PAGE using 7% gels. Proteins were visualized by Colloidal Coomassie Brilliant Blue staining or Western Blotting. Western Blotting was performed using a tank blot system (BioRad) and proteins were detected with a primary anti-His antibody (Qiagen), anti-PDR5 antibody (Davids Biotechnologie, Regensburg, Germany) or a C219-antibody (Abcam) in combination with a secondary anti-mouse antibody or anti-rabbit antibody, respectively, labeled with horseradish peroxidase (Jackson Immuno Research). Protocols followed the manufacturer’s instructions.

## Results

### Cloning of ABC transporters of the PDR family

For expression studies in *P*. *pastoris* the full-size ABC transporters *At*ABCG30/PDR2, *At*ABCG32/PDR4, *At*ABCG33/PDR5, *At*ABCG34/PDR6, *At*ABCG35/PDR7 and *At*ABCG36/PDR8 were chosen, all of which belong to the *A*. *thaliana* PDR family. These proteins are 157 to 165 kDa in size and contain 10 to 14 predicted transmembrane helices [66]. The coding sequences from *A*. *thaliana* cDNA were amplified in order to clone the genes into pJET1.2 for sequencing and subsequent cloning into the expression vector. Likely due to the large gene sizes (4.2–4.7 kbp), PCR protocols had to be optimized for each individual gene, including a screening for the correct annealing temperature combined with testing different polymerases and various buffer conditions. *PDR6* was successfully amplified using Phusion DNA polymerase (NEB), however concentrations of the resulting PCR product was so low that additional PCRs of the first product were performed. Alternatively, *PDR5* and *PDR8* were amplified with PrimeStar GXL DNA polymerase (Clonetech). Despite various efforts, the complete coding sequences of *PDR2*, *PDR4* and *PDR7* could not be amplified in one step from the cDNA. Therefore, these three genes were amplified using GXL DNA polymerase in two fragments subsequently and cloned separately as described below. All the resulting PCR products were blunt-end ligated into pJET1.2, thereafter amplified in *E*. *coli* and subsequently sequenced prior to subcloning into the expression vectors.

### Subcloning into pSGP18-2μ *P*. *pastoris* expression vector

In order to clone constructs for expression in *P*. *pastoris* different cloning strategies were employed. Since the complete coding sequences of PDR5, PDR6 and PDR8 were present as a single fragment, restriction digestion and ligation cloning was used for these genes. Using this, PDR6 and PDR8 were successfully cloned into pSGP18-2μ vector with a CBP tag followed by a hexa-histidine (His_6_) tag at the C-terminus of the protein sequence. However, in order to find the correct positive clone, at least 30 colonies were screened per transformation. In addition, the number of colonies on plates was very low, of which most contained just a fragment of the gene or were false positives. In the case of PDR5, restriction digestion and ligation cloning was unsuccessful.

Alternatively, In-Fusion cloning was used for the genes that existed in two separate fragments in pJET1.2, so that both parts are combined in the pSGP18-2μ expression vector. Using standard protocols, either none or very few colonies were obtained, which did not grow in liquid culture medium or did not contain the full plasmids as extensive parts of the gene were missing. The subsequent step was to either try alternative cloning strategies or optimize the In-Fusion cloning protocol.

Thus, homologous recombination was employed as an alternative cloning strategy. In addition to the 2μ ori present in the *P*. *pastoris expression* vector pSGP18 [44], which allows homologous recombination in *S*. *cerevisiae*, λ-Red homologous recombination in *E*. *coli* [68] was also used. Both homologous recombinations resulted mostly in the absence of positive clones. In the case of pSGP18-2μ-*PDR7*, some positive clones were however obtained. The isolated plasmids could not be re-transformed into *E*. *coli* despite testing different strains (DH5α, XL1-Blue, XL10-Gold NEB Turbo, SURE 2), incubation temperatures (37°C, 30°C, RT) and media for regeneration. In addition, the yield of plasmid was low and not sufficient for direct transformation into *P*. *pastoris*. The λ-Red homologous recombination of the two fragments of *PDR4* in *E*. *coli* resulted in a few positive clones, however, inoculation of these clones in liquid cultures led to very poor or no cell growth at all. Since this approach was not promising, it was not used further for the remaining constructs. Instead, the In-Fusion protocol was optimized by performing gel-extraction of the DNA fragments, doubling the incubation time and changing the incubation temperature of transformed bacteria to 30°C in comparison to the previously used temperature of 37°C. Despite the above-mentioned strategies, numerous colonies had to be screened in order to observe positive clones. After these extensive efforts, the constructs PDR2 and PDR7 in pSGP18 were successfully obtained. However, it is worth mentioning here that, throughout the cloning process, frequent point mutations occurred in the sequences that were rectified by site-directed mutagenesis PCR.

### Expression of PDR transporters in *P*. *pastoris*

The successfully cloned constructs pSGP18-2μ-PDR8, pSGP18-PDR6, pSGP18-PDR2 and pSGP18-PDR7, respectively, were tested for small-scale expression in *P*. *pastoris* by selecting cells carrying the transformed genes from their respective antibiotic containing agar plates. The transformation was verified by colony PCR or sequencing the DNA obtained after plasmid isolation.

First, constructs were tested for expression using the standard protocols (Invitrogen). Since protein expression could not be detected, culture conditions were optimized. Buffered complex media were used instead of minimal media. Different incubation temperatures were tested ranging from 25 to 30°C. Additional pre-cultures were made to obtain higher cell density before the main culture was set up. In order to increase the oxygen level in the flasks, the cultivation volume of 50 ml was also reduced to 25 ml. Additionally, to obtain a better initial mRNA-ribosome interaction, the codon usage of very rare codons within the first 100 amino acids (which were all coding for arginine) was optimized for expression in *P*. *pastoris*. This can add to the stability of the initiation complex and lead to an improved protein expression [76]. However, all these extensive efforts did not result in expression in *P*. *pastoris*.

### Subcloning: Change of tag position

Since no expression was detected for C-terminally tagged PDR transporters in *P*. *pastoris*, the localization of the tag might be crucial for expression in *P*. *pastoris*. In order to check this hypothesis, N-terminally tagged constructs for expression in *P*. *pastoris* were generated. For this purpose, the affinity tags were synthetically produced containing a deca-Histidine (His_10_) and a CBP sequence (GeneArt Gene Synthesis, Invitrogen). Via In-Fusion reaction, the N-terminal tag sequence was added to the pSGP18 vector, which was previously amplified without the C-terminal tag. A codon-optimized sequence of PDR2 was synthetically produced and cloned into pSGP18-Ntag by Genscript, USA. Remarkably, during the cloning process, the company reported various instances of gene instability and re-occurring mutations. Apart from screening numerous clones, we like to stress that the problems were solved by using CopyCutter^TM^ EPI400^TM^ *E*. *coli* cells for the cloning process, which were consequently integrated into our subsequent cloning protocols. Using the above-mentioned strategy pSGP18-PDR8-Ntag was successfully obtained using In-Fusion cloning.

### Expression of N-terminally tagged PDR transporters in *P*. *pastoris*

**Fig 1:**
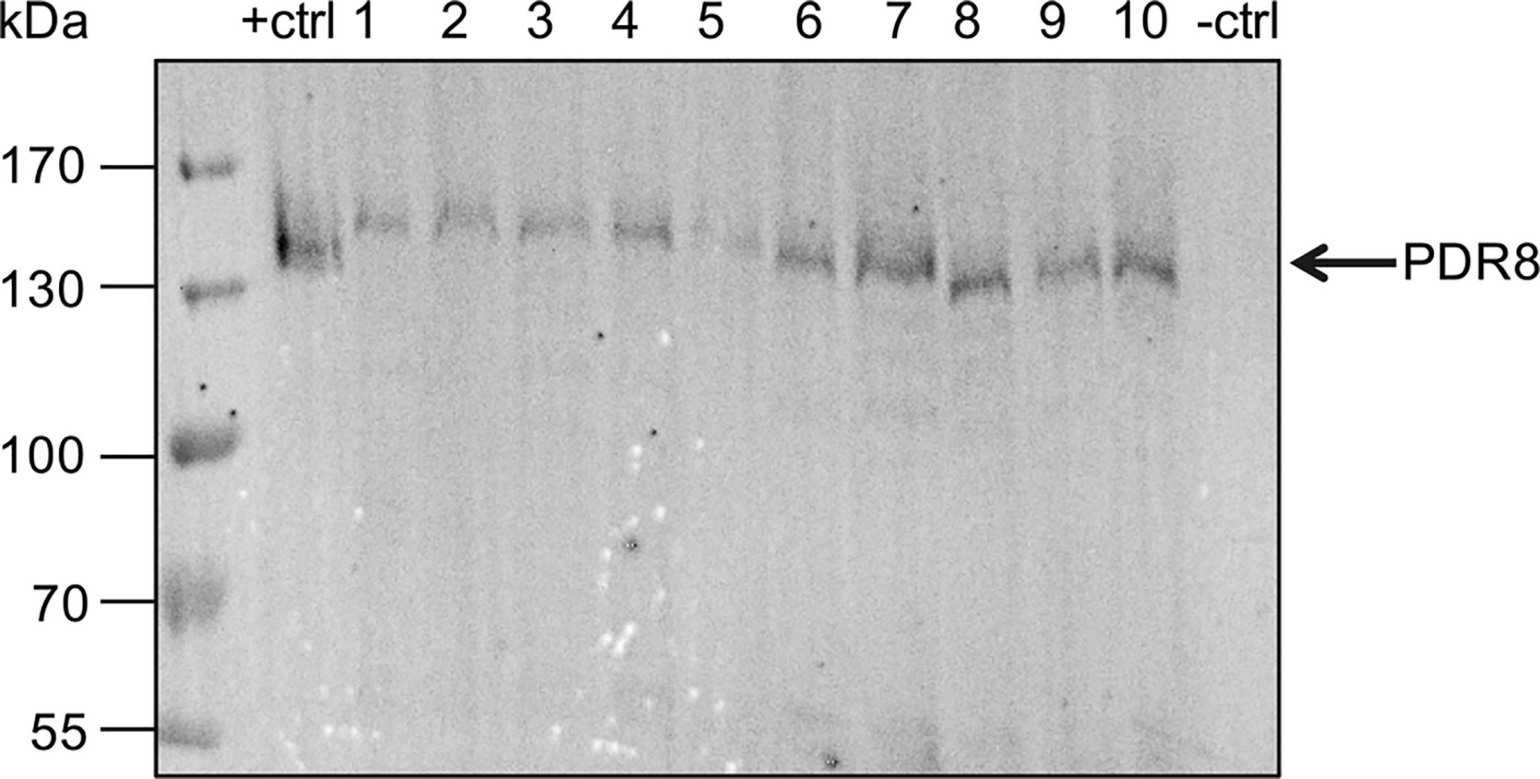
Heterologous expression of PDR8 in *P*. *pastoris*. Crude membranes derived from *P*. *pastoris* cells carrying pSGP18 (- ctrl: empty vector), pSGP18-2μ-BSEP expressing the Bile Salt Export Pump [44] (+ ctrl: positive control) and pSGP18-PDR8-Ntag clones (1–10) were analyzed via SDS-PAGE and immunoblotting (anti-His-tag antibody).

**Fig 2:**
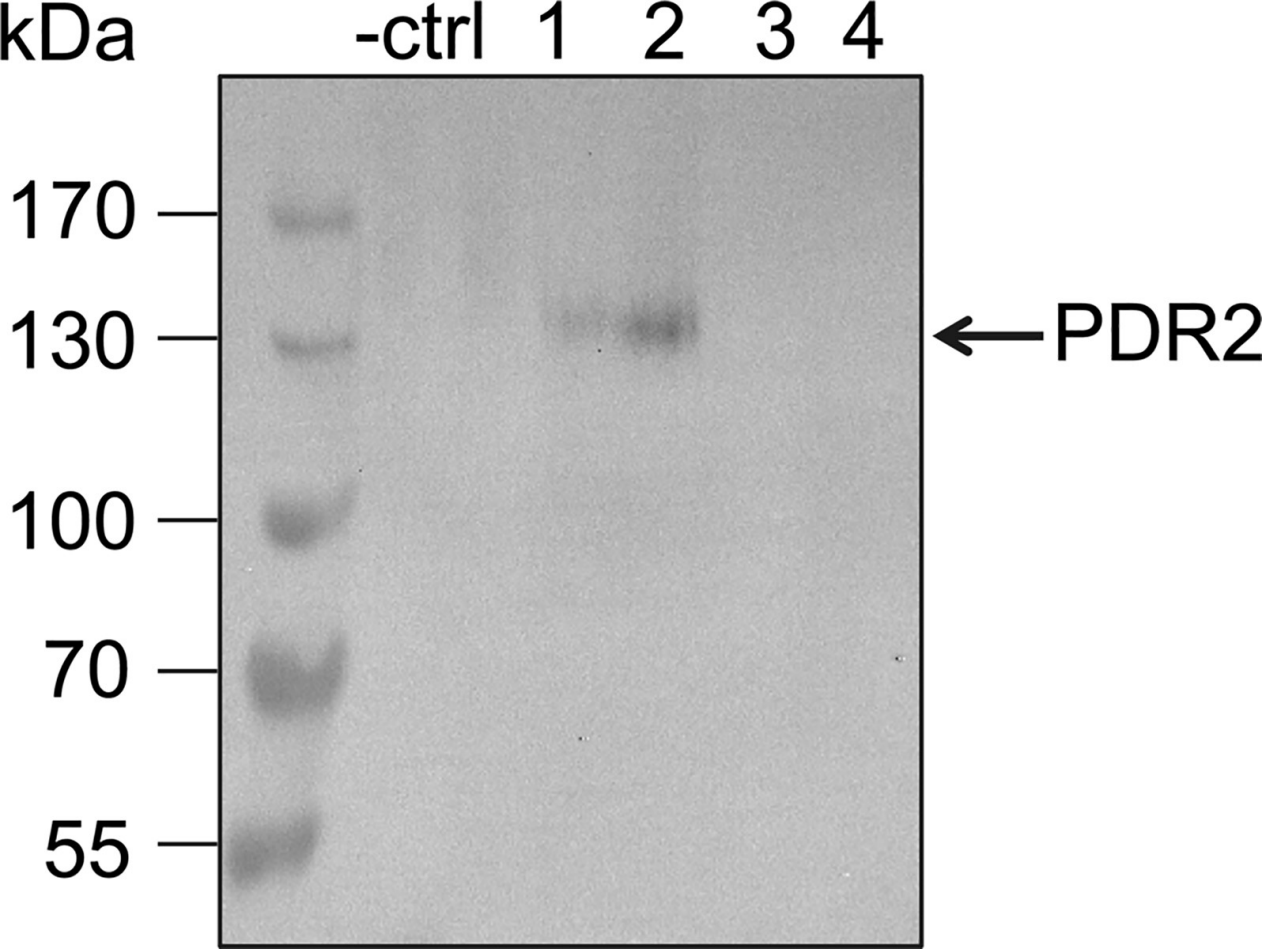
Heterologous expression of PDR2 in *P*. *pastoris*. Crude membranes derived from *P*. *pastoris* cells carrying pSGP18 (-ctrl: empty vector), and pSGP18-PDR2-Ntag clones (1–4) were analyzed via SDS-PAGE and immunoblotting (anti-His-tag antibody).

Initial expression trials of N-terminally tagged PDR2 and PDR8 in *P*. *pastoris* resulted in expression of truncated proteins as confirmed via Western Blot analysis (not shown). Additional sequencing of the constructs revealed that during propagation in *E*. *coli*, a stop codon was created in both protein sequences, although the sequences of the constructs were correct after cloning. In another instance, the re-transformed *E*. *coli* clone lacked the start codon. Therefore, it is necessary to verify the sequence after every re-transformation step. After sequence rectification, the N-terminally tagged constructs pSGP18-PDR2 and pSGP18-PDR8 were successfully expressed in *P*. *pastoris* using standard protocols. To select for multiple copy integrations of the respective gene in the expression host, clones were subjected to increasing zeocin concentrations, as it was done by Chloupkova et al. [60]. Protein expression was obtained for all tested PDR8 clones and clone 7 was used for further studies (Fig 1). Out of the four tested PDR2 clones two clones showed expression as confirmed by Western Blot analysis. Here, clone 2 was subsequently used (Fig 2). For the PDR2 and PDR8 constructs, the position of the tag was crucial for successful overexpression.

### Cloning and expression of WBC1: half-size meaning half-problems?

**Fig 3:**
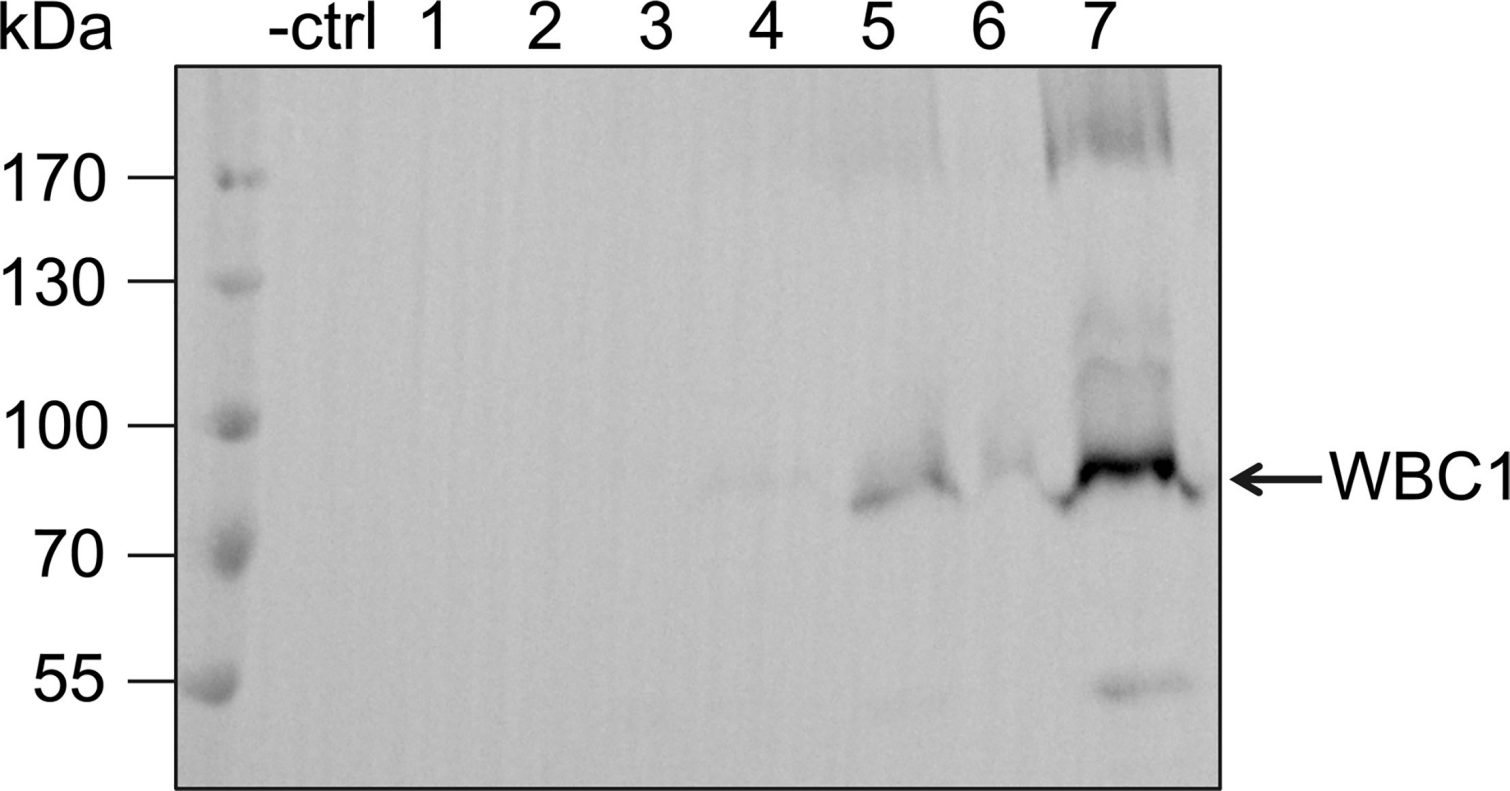
Heterologous expression of WBC1 in *P*. *pastoris*. Crude membranes derived from *P*. *pastoris* cells carrying pSGP18 (- ctrl: empty vector), and pSGP18-WBC1-Ntag clones (1–7) were analyzed via SDS-PAGE and immunoblotting (anti-His-tag antibody).

Bernaudat *et al* (2011) screened for expression of different membrane proteins using different expression systems [39]. They reported that successful overexpression highly depends on the size of the protein and the number of transmembrane helices. This raised the possibility that vector construction and expression was only challenging and extremely time consuming for the full-size PDR transporters of the ABCG family. Therefore, we cloned *WBC1*, a half-size transporter of the ABCG family. In these transporters, only a single NBD and a single TMD are encoded on the gene and the protein has to dimerize to form a functional transporter. Subsequently, the protein, with a molecular weight of 82 kDa, is smaller than the full-size PDR transporters mentioned above. The gene was produced synthetically and codon-optimized for expression in *P*. *pastoris* (Invitrogen) and afterwards cloned via In-Fusion into pSGP18-Ntag vector. In addition, *WBC1* was also amplified from cDNA without any problems. The cloning of the half-size transporter *WBC1* was easily performed compared to the problematic cloning of the PDRs, clearly indicating that the large gene size was one reason of the complicated cloning of the full-size transporters. Furthermore, none of the problems mentioned above were observed. As shown in Fig 3, small-scale expression studies of pSGP18-WBC1-Ntag resulted in three positive clones out of seven clones tested. The bands of approximately 80 and 160 kDa, respectively, clearly indicate that WBC1 was detected in the monomeric, as well as the dimeric state. Clone 7 was used for further studies.

### Subcellular localization of PDR2, PDR8 and WBC1

**Fig 4:**
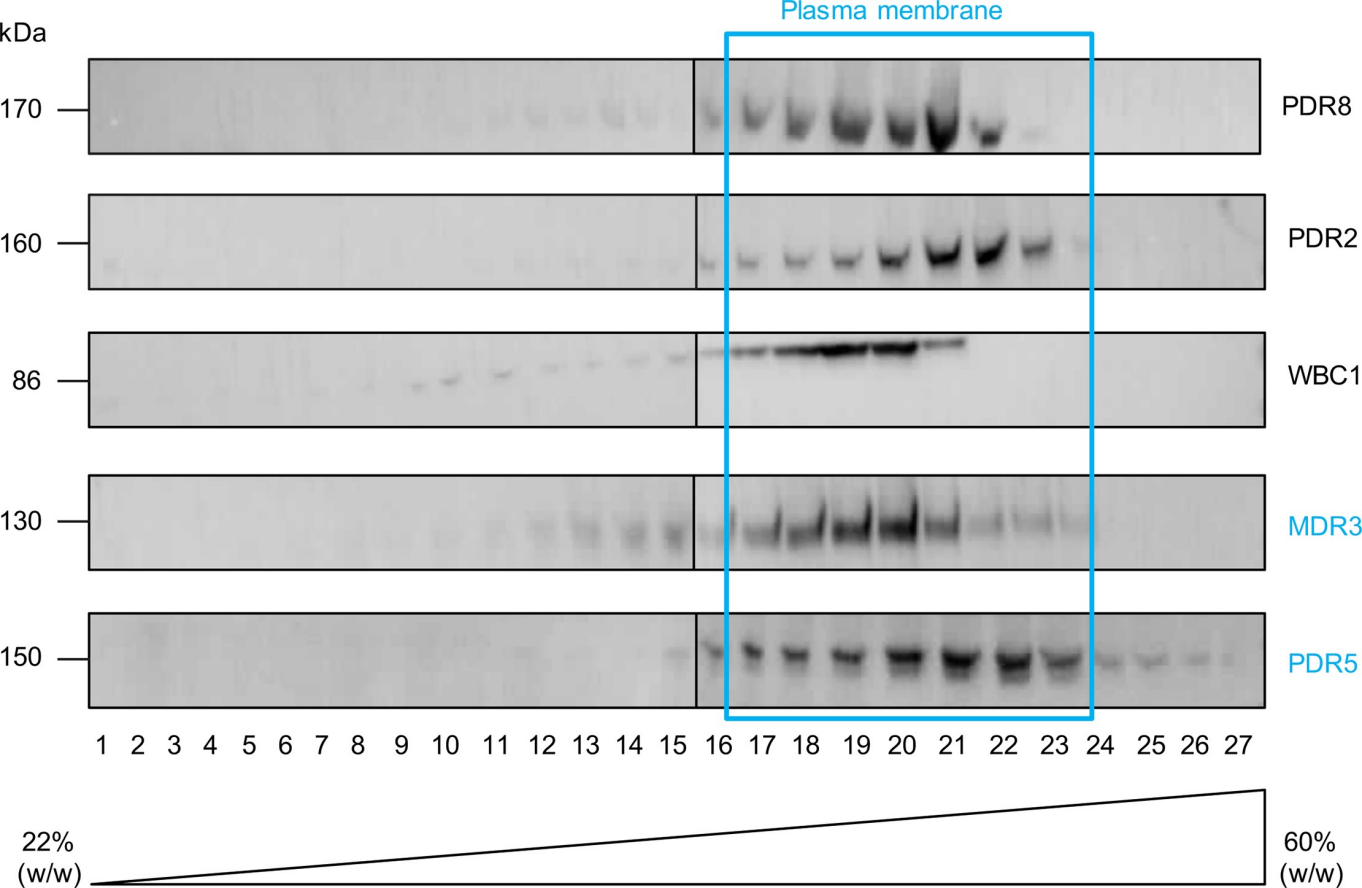
Sucrose gradient centrifugation of PDR8, PDR2 and WBC1 in *P*. *pastoris*. *P*. *pastoris* crude membranes expressing WBC1, PDR8, PDR2 and MDR3 and *S*. *cerevisiae* crude membranes expressing PDR5 were separated via ultracentrifugation through a continuous sucrose gradient. Samples were analyzed by SDS-PAGE and immunoblotting (anti-His-tag antibody, anti-PDR5 antibody, C219 antibody). The analyzed proteins are co-localized with the plasma membrane proteins MDR3 and PDR5. The displayed results were assembled from different Western Blots. Entire blots can be found in the supplemental material (S1–S10 Figs).

**Fig 5:**
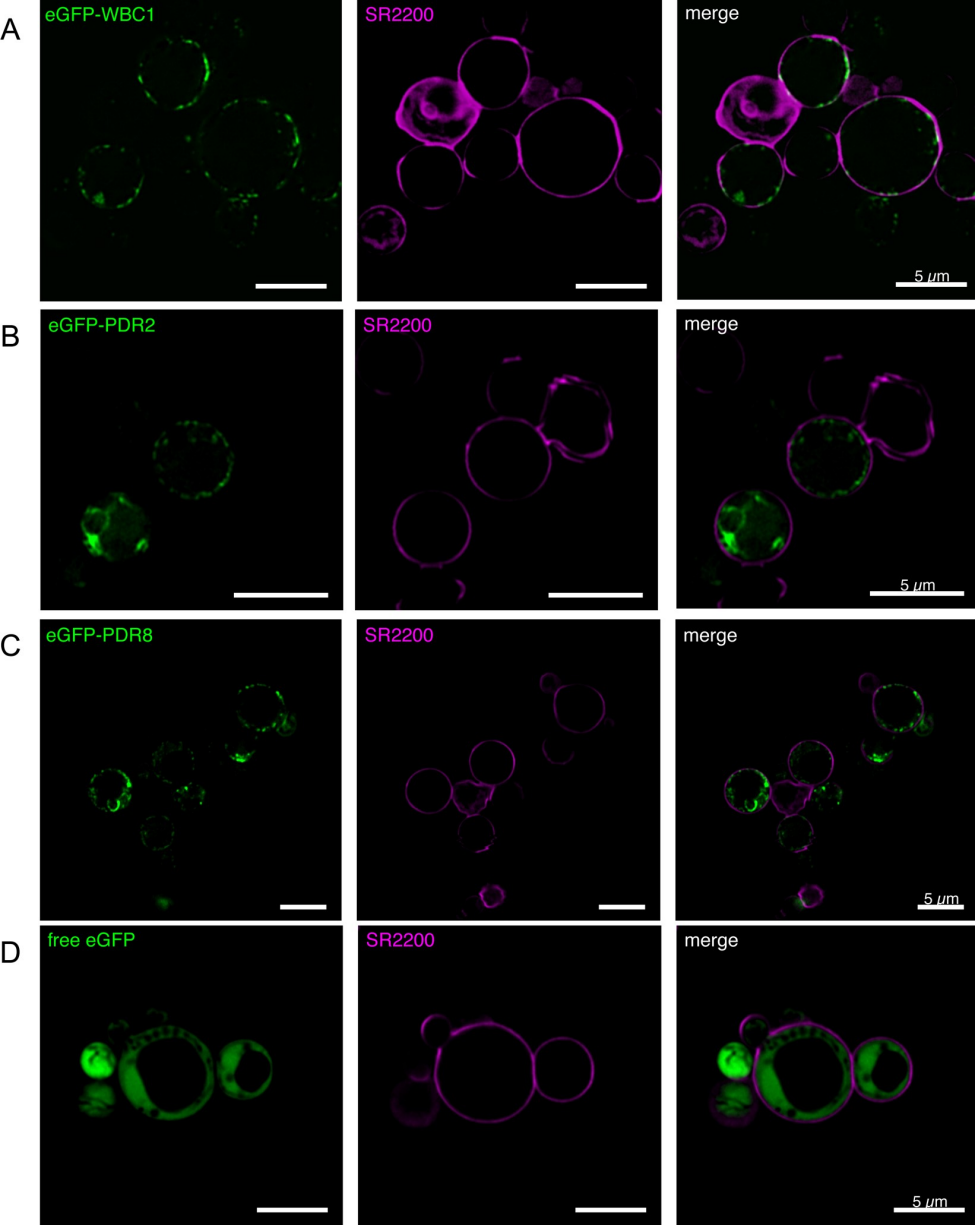
Confocal fluorescence microscopy of eGFP-PDR8, eGFP-PDR2 and eGFP-WBC1 in *P. pastoris*. *P*. *pastoris* cells expressing either the fusion proteins eGFP-WBC1 (row A), eGFP-PDR2 (row B), or eGFP-PDR8 (row C) were analyzed by confocal fluorescence microscopy. As a control cells expressing free eGFP (row D) were analyzed as well. The left lane shows the eGFP fluorescence signal of the respective fusion protein, the middle lane the SR220 signal and the right lane the merged signals.

**Fig 6:**
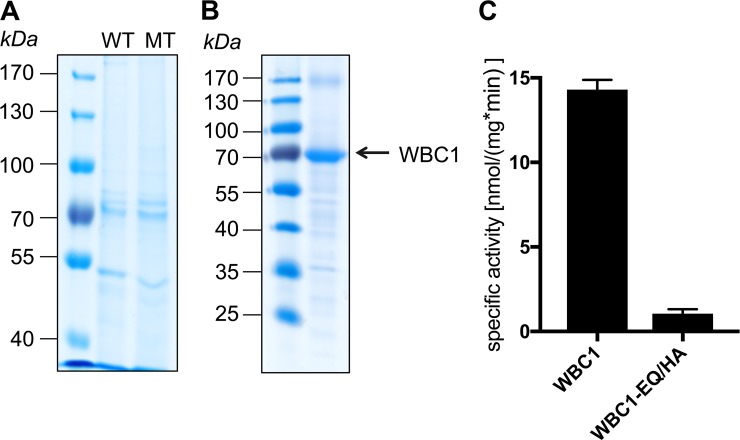
Basal activity of purified WBC1. Wild type WBC1 (WT) and the EQ/HA mutant (MT) were purified via CBP-purification and analyzed subsequently by SDS-PAGE and Colloidal Coomassie Brilliant Blue staining (A). B shows the concentrated WBC1 fraction. Basal activity of purified wild type WBC1 and the EQ/HA mutant was determined by measuring the specific activity (C). Data represents mean and ± SD of at least three independent replicates.

In order to determine the localization of the two PDRs and WBC1 overexpressed in *P*. *pastoris* a continuous sucrose gradient centrifugation was performed. As shown by Western Blot analysis (Fig 4), the three proteins were detected in the gradient in the same fractions as the plasma membrane protein Pdr5 from *S*. *cerevisiae* [72] and as human MDR3, which was successfully expressed in *P*. *pastoris* before and localized to the plasma membrane [44, 72]. In addition to this, the fusion proteins eGFP-PDR2, eGFP-PDR8 and eGFP-WBC1 were expressed in *P*. *pastoris*. The additional eGFP tag at the N-terminus reduced the expression levels of all three proteins so that detection by immunoblotting was not possible (data not shown). However, confocal fluorescence microscopy could confirm that the three fusion proteins were expressed (Fig 5). To mark the cell wall the *P*. *pastoris* cell walls were stained with the marker SCRI Renaissance 2200 (SR2200). The eGFP signal of the eGFP-WBC1 fusion protein clearly shows a ring-like signal that almost overlaps with the SR2200 signal indicating that the eGFP-WBC1 fusion protein localized to the plasma membrane (Fig 5, row A). However, such clear result was not obtained for the PDR fusion proteins. The eGFP signal was found for the majority of the cells in membrane structures inside the cells, while only in few cells PDR2 (Fig 5, row B) and PDR8 (Fig 5, row C) seem to localize in the plasma membrane with very faint eGFP-signals that formed rings around the cells. This indicates that in most cells the eGFP-PDR fusion proteins were still in located in endomembrane compartments. As a control free eGFP was expressed as well, which resided in the cytoplasm of *P*. *pastoris* cells. The GFP signal can be clearly distinguished from the SR2200 signal (Fig 5, row D). Compared to these results, *P*. *pastoris* cells without heterologously expressed protein did not fluoresce (data not shown). Taken together, WBC1 was the only transporter which was shown by sucrose gradient as well as confocal microscopy to be correctly targeted in the plasma membrane. Hence, WBC1 and an ATPase hydrolysis deficient mutant WBC1-EQ/HA were purified by calmodulin binding peptide affinity chromatography from *P*. *pastoris* crude membranes. Subsequent ATPase assays exhibited a basal activity 13.26 ± 0.32 nmol/min per mg purified WBC1 (Fig 6).

## Discussion

Compared to other organisms, plants exhibit a large variety of membrane transporters, with over 100 genes encoding for plant ABC proteins. We are focusing on the ABCG subfamily of which many have been analyzed regarding their function and expression [8, 11, 77–79]. However, identification of transported substrates is often complicated by their pleiotropic effects and reports on purified and biochemically analyzed proteins are still rare. We selected eight PDR transporters for heterologous expression. Until now, plant PDR transporters have been successfully expressed for example in yeast systems *S*. *cerevisiae*, *S*.*pombe* [80], insect cells [81] or in BY2 plant suspension cultures [65, 82].

Here, we evaluate the methylotrophic yeast *P*. *pastoris* as an alternative expression system for plant PDR transporters. As shown in previous studies, *P*. *pastoris* is a suitable host for heterologous expression of membrane proteins [44, 60, 83]. Major advantages include tightly regulated promoters such as the alcohol oxidase (*AOX1*) promoter [84] and fermentable cells, which can reach high cell densities. Fed batch fermentation of successfully cloned plant PDR transporters lead up to 150 g wet cells per liter. Thus, even proteins with limited cell expression per single cell, can be produced in substantial amounts.

First cloning attempts were complicated for instance by difficulties in cDNA amplification or re-occurring mutations. To rule out that these difficulties derived from the used expression vector, additional vectors for expression in different host systems, including the yeast *S*. *cerevisiae* and bacterial systems *E*. *coli* and *L*. *lactis*, were selected. When we compared cloning results for the different expression vectors, cloning into *P*. *pastoris* vectors seemed more achievable (data only shown for *P*. *pastoris*). Hence it was decided to focus on optimization of cloning into *P*. *pastoris* vectors. So far, detailed reports about difficulties encountered while cloning of unstable or toxic DNA are rare. Therefore, critical factors which affected the cloning and various solution approaches will be highlighted in the following section.

### Cloning of plant PDR transporters

One basic step in cloning is the amplification of the DNA. In our case, the amplification of the selected *PDR* genes from the cDNA of *A*. *thaliana* emerged as the first hurdle. Out of 8 PDR genes only PDR8 and PDR6 were fully amplified in one step, while the remaining genes could only be amplified in two fragments. The subsequent step included a two-step cloning. At first, the genes were cloned into a cloning vector and subsequently sequenced and amplified into the respective expression vectors. The first step of the cloning process often resulted in empty vectors or wrong inserts, although the size of the PCR product was always verified by gel electrophoresis. Hence, successful cloning of the gene into the cloning vector not only required the usual gene-specific optimization of the PCR reaction, but also screening of many clones. In the second step of cloning, the genes or gene fragments were cloned from the cloning vector into the vectors for heterologous expression. Different techniques, such as homologous recombination in *E*. *coli* and *S*. *cerevisiae* [68, 69], classical cloning via restriction sites or In-Fusion cloning were used for this purpose. Similar to the initially performed cloning reactions using cDNA, most reactions resulted either in empty vectors, mutations or significantly reduced number of clones. In addition to that, poor cell growth was observed after retransformation. One important aspect to consider here is that cloning depends on many factors, such as for example vector properties or gene toxicity. In the case of gene toxicity, a leaky promoter could be lethal for the cells [85]. Every promoter has basal activity, which in combination with a high copy plasmid can lead to the accumulation of toxic proteins within the cell [86], resulting in poor cell growth and / or plasmid instability. Additionally, the cells could be more inclined to mutate the genes in order to circumvent gene toxicity. Hence, toxic genes can affect the cloning, plasmid stability and protein expression.

In this study, cloning into the expression vector was mainly performed using *E*. *coli* and our attempts to express PDR8 heterologously in *E*. *coli* failed due to reoccurring stop codons resulting in truncated proteins (data not shown). After mutating these stop codons to the proper coding sequence, another stop codon appeared at a different position of the gene. It was also observed that after transformation of *E*. *coli*, the start codon of the *PDR* sequence was deleted, though sequencing results confirmed that the PCR products used in the transformation steps were correct. However, similar results had been obtained for NpPDR1 overexpression in *Nicotiana tabacum*. The cloning process had failed because the gene was always mutated during cloning in *E*. *coli* [19]. Thus, it is very likely that *E*. *coli* cannot propagate the construct of the full-size PDR genes due to toxicity. Poor cell growth could be ascribed to the fact that maintenance of a eukaryotic, and probably toxic gene, could require more metabolic energy. Thus, in order to counteract this phenomenon, various competent cell strains were used besides the standard strains such as XL1-Blue, XL10-Gold or DH5α. Strains such as CopyCutter EPI400, NEB Turbo and SURE 2 were also used, which are specifically designed for cloning of unstable or toxic DNA. SURE 2 cells lack genes, which are involved in rearrangement and deletion of unstable DNA structures in *E*. *coli*, while CopyCutter cells are able to lower the number of many popular high-copy number plasmids. Since using low copy number plasmids can lower the basal activity, and thereby the expression of likely toxic gene products can be limited [87]. As seen from our results, CopyCutter cells seemed to be a better choice for this purpose. This indicates that the selected PDR genes were difficult to clone into *E*. *coli* due to toxicity. Furthermore, we experienced that cell cultivation at lower temperatures and prolonged incubation times improved very often the chances for positive clones.

After extensive screening of clones and optimization of every cloning step, it was finally possible to clone a number of genes successfully for expression. While the *P*. *pastoris* expression constructs pSGP18-PDR2-Ctag, pSGP18-PDR6-Ctag, pSGP18-PDR7-Ctag and pSGP18-PDR8 with tag at both the terminus were cloned in our labs, pSGP18-PDR2-Ntag and pSGP18-WBC1-Ntag were synthesized. On the contrary, an attempt to clone the half-size transporter *WBC1* from cDNA was straightforward. This clearly indicates that cloning of full-size PDR plant ABC transporters is quite challenging, and many factors such as gene size, cDNA quality, vector properties and gene toxicity for the host cell play an important role in the success of cloning.

### Heterologous expression of plant PDR transporters

To date, only a few *A*. *thaliana* PDR transporters such as PDR1, PDR2 [25], PDR3 [64], PDR6 [82] PDR9 [27] and PDR12 [33] have been heterologously expressed. Hence, it is of great importance to analyze if *PDR* gene expression is complicated due to the nature of the gene, the expression host or tagging site.

As already described above, cloning of plant *PDR* genes can be quite difficult and time consuming. However, a subset of the intended expression constructs were successfully cloned in *P*. *pastoris* and expression studies were subsequently performed. Although most of the N-terminally tagged constructs (PDR2, PDR8, WBC1) were expressed. Fig 1 shows a variation in expression level between the respective PDR8 clones. This might be due to varying copy numbers of the PDR8 gene in the *P*. *pastoris* genome. It is a well known phenomena that *P*. *pastoris* is able to integrate transformed DNA multiple times in its genome. Plating clones on high zeocin concentrations ensured the selection of multiple copy clones [88].

Contrary to this, all C-terminally tagged proteins did not express. Additionally, the codon usage for rare arginine codons within the first 100 amino acids of the protein sequence was also optimized for PDR7 and PDR2 (C-tagged constructs) to improve the stabilisation of the initiation complex as shown previously by Chen and Inouye [76]. Neither by using a His-antibody nor a CBP-antibody was it possible to detect any protein expression for the C-terminal tagged constructs. In accordance with that, it has been shown that the tag position can affect the yield of protein [89]. Thus the question arises if the protein was expressed at all or if the tag was inaccessible for immunoblot verification.

A possible reason for the lack of detectable expression could be the secondary structure of the mRNA. It has been previously shown that mutations within the ribosome binding site (rbs) lead to hairpin stability change and thereby affect the translation efficiency in *E*. *coli* [90]. Specific structures, such as stem-loops, can even lead to a blockage of the rbs, thus, inhibiting translation [91–93]. Notably there are studies indicating that positioning the tag at the amino-terminus can destabilise secondary structures, such as hairpin loops at the rbs, and hence, facilitate translation [94, 95]. In summary, our results indicate that N-terminal tagging leads to protein expression of plant ABC transporters of the PDR family. This is demonstrated by the expression tests of PDR8, where only the re-positioning of the tag was aquired to achieve successful overexpression. In addition to this, codon optimization, which includes the codon usage optimization but also the adjustment of the GC content, prevention of stem-loop formation and cis-acting sites, might support successful overexpression.

### Localization of heterologously expressed proteins in *P*. *pastoris*

For subsequent functional studies, the proper cellular localization of the overexpressed ABC transporters is of great importance in the heterologous system. *In planta* PDR8 is localized to the plasma membrane of leaf epidermal cells, as well as to the periphery of epidermal and lateral root cap cells [96, 97]. PDR2 was shown to be located in the plasma membrane of mesophyll protoplasts [33] and WBC1 localizes to the plasma membrane when transiently expressed in *N*. *benthamiana* [98].

In our first attempt for subcellular protein localization in *P*. *pastoris*, it was evident that WBC1, PDR2 and PDR8 localize in the plasma membrane. The proteins were mainly detected in the same fractions as the two membrane proteins that were known to localize to the plasma membrane, Pdr5 and MDR3 (Fig 4). Nevertheless, further faint protein bands were detected almost in the entire gradient and therefore we decided to verify the plasma membrane localization by confocal microscopy with eGFP-fusion proteins of PDR2, PDR8 and WBC1.

The fluorescence microscopy of the eGFP fusion proteins in *P*. *pastoris* cells showed a distinct plasma membrane localization for WBC1. In contrast to that, PDR2 and PDR8 were only partially located in the plasma membrane and mainly seen in other compartiments (Fig 5). This could correlate with the protein size and / or the number of transmembrane helices. WBC1 is a half-size transporter and therefore it is possible that it passes the protein control faster than the full-size transporters. PDR2 and PDR8 probably get stucked during the protein processing and hence are not completely targeted to the plasma membrane. In support of this, the overall protein expression level of WBC1 in *P*. *pastoris* has been even after fermentation experiments (data not shown) higher in comparison to PDR2 and PDR8. Similar observations, have been previously made by Bernaudaut *et al*., who postulated that increasing numbers of amino acids or transmembrane helices impede the heterologous expression of membrane proteins [39].

Another possible reason for the partial mistargeting could be that the eGFP-fusion site can lead to mistargeting as already shown for the full-size human ABC transporter ABCC2 [99]. Drew et al. showed that C-terminal tagging facilitates the correct localization [100], while others reported mistargeting [99]. It is therefore generally recommended to test both C-terminal and N-terminal eGFP-fusion constructs. But in our case it is not appliable, because C-terminal His-tagging exhibited no protein expression and hence a C-terminal GFP-tag would lead most probably to the same result. Taken together, the plasma membrane localization of WBC1, PDR2 and PDR8 in *P*. *pastoris* was proven via sucrose gradient centrifugation, while a verification via confocal microscopy was only possible for WBC1. However, we believe that partial mistrafficking of PDR2 and PDR8 in in the later part was likely a consequence of decreased protein expression due to size and GFP-fusioning. Subsequent initial functional studies with WBC1 exhibited a basal activity 13.26 ± 0.32 nmol/min per mg purified WBC1 in presence of 5 mM ATP. Other ABCG transporters like NpPDR5, that was purified from a plant expression system or NtPDR1, that was purified from BY-2 expression cells displayed v_max_ (maximal basal activity) of 27.0 ± 5.2 nmol/min/mg and 21.2 ± 2.4 nmol/min/mg, respectively [101]. If WBC1 is able to reach similar v_max_ values has to be analyzed in future studies. However, this indicates the suitability of *P*. *pastoris* as alternative expression system for plant half-size transporters. Similar studies need to be performed in order to analyze the functionality of *P*. *pastoris* expressed PDR2 and PDR8.

### Conclusions

Although cloning of plant PDR transporters can be quite laborious and seems unattainable, it is feasible provided the following points are taken into account. Our results clearly indicate that the amplification from cDNA is the first critical and crucial step. Therefore, it is important to localise the parts of the plants and conditions wherein the respective genes are highly expressed, and then subsequently obtain the cDNA.

In case the usual optimization of the PCR does not work, the gene should be amplified in two or more fragments, although it complicates the subsequent cloning steps. Furthermore, it is worth spending more time on method optimization and extensive screening for positive clones. In the present study, optimization of In-Fusion and/or classical cloning lead to successful cloning of some of the selected *PDR* genes (*PDR2*, *PDR6*, *PDR7* and *PDR8*). Thus, spending more time on method optimization and extensive screening for positive clones should facilitate cloning of any *PDR* gene into any vector. In addition, using CopyCutter cells or comparable strains that supress plasmid replication as cloning host should be highly advantegous. Even after successful insertion of the *PDR* gene into the expression vector, frequent mutations in the coding sequence occurred, which led to insertion of stop codons or deletions of start codons. In such cases, it is worthwhile to perform a site-directed mutagenesis PCR as it is easier to re-mutate the sequence than restarting the entire cloning process.

The present study demonstrates the possible difficulties and the corresponding solutions to successful cloning of plant PDR genes. Beyond that, the methylotropic yeast *P*. *pastoris* is introduced and evaluated here for the first time as heterologous expression host for plant PDR transporters. It has the advantage that fermentation processes can lead to large biomasses, which can compensate the low expression rats of poorly produced membrane proteins. In this study the position of the tag at the N-terminus was crucial for successful overexpression while no expression was detected for C-tagged proteins. In addition, sequence optimization regarding the codon usage and other elements that influence mRNA stability can contribute to successful overexpression of heterologous proteins. As our results indicate, cloning and expression of plant PDR transporters can emerge to be a delicate process. It is therefore advisable to keep the options broad with for example different fusion proteins and tagging sites.

We were able to express constructs of up to 170 kDa in sufficient amounts for subsequent biochemical studies. Hence, we introduce here *P*. *pastoris* as an alternative option for heterologous expression of plant ABC transporters. Initial functional studies with WBC1 showed basal activity, which can be analyzed for instance in future studies for substrate-stimulation. Moreover, until now *atpdr2* and *atpdr8* mutant studies exhibited pleiotropic effects in plants [16, 30, 33, 36–38]. As shown by sucrose gradient centrifugation PDR2 and PDR8 are correctly targeted in the plasma membrane of *P*. *pastoris*. Hence, this study provides a proper foundation for further functional studies, which could help to clarify the roles of this potential phytohormone transporters.

## Supporting information

S1 Fig Sucrose gradient fractions 1 to 15 of PDR8 containing *P*. *pastoris* crude membranes. Crude membranes were separated via ultracentrifugation through a multistep sucrose gradient. The samples were analyzed by SDS-PAGE and immunoblotting (anti-His-tag antibody).
(TIF)

S2 Fig Sucrose gradient fractions 16 to 27 of PDR8 containing *P*. *pastoris* crude membranes. Crude membranes were separated via ultracentrifugation through a multistep sucrose gradient. The samples were analyzed by SDS-PAGE and immunoblotting (anti-His-tag antibody).
(TIF)

S3 Fig Sucrose gradient fractions 1 to 15 of PDR2 containing *P*. *pastoris* crude membranes. Crude membranes were separated via ultracentrifugation through a multistep sucrose gradient. The samples were analyzed by SDS-PAGE and immunoblotting (anti-His-tag antibody).
(TIF)

S4 Fig Sucrose gradient fractions 16 to 27 of PDR2 containing *P*. *pastoris* crude membranes. Crude membranes were separated via ultracentrifugation through a multistep sucrose gradient. The samples were analyzed by SDS-PAGE and immunoblotting (anti-His-tag antibody).
(TIF)

S5 Fig Sucrose gradient fractions 1 to 15 of WBC1 containing *P*. *pastoris* crude membranes. Crude membranes were separated via ultracentrifugation through a multistep sucrose gradient. The samples were analyzed by SDS-PAGE and immunoblotting (anti-His-tag antibody).
(TIF)

S6 Fig Sucrose gradient fractions 16 to 27 of WBC1 containing *P*. *pastoris* crude membranes. Crude membranes were separated via ultracentrifugation through a multistep sucrose gradient. The samples were analyzed by SDS-PAGE and immunoblotting (anti-His-tag antibody).
(TIF)

S7 Fig Sucrose gradient fractions 1 to 15 of MDR3 containing *P*. *pastoris* crude membranes. Crude membranes were separated via ultracentrifugation through a multistep sucrose gradient. The samples were analyzed by SDS-PAGE and immunoblotting (C219 antibody).
(TIF)

S8 Fig Sucrose gradient fractions 16 to 27 of MDR3 containing *P*. *pastoris* crude membranes. Crude membranes were separated via ultracentrifugation through a multistep sucrose gradient. The samples were analyzed by SDS-PAGE and immunoblotting (C219 antibody).
(TIF)

S9 Fig Sucrose gradient fractions 1 to 15 of PDR5 containing *S*. *cerevisiae* crude membranes. Crude membranes were separated via ultracentrifugation through a multistep sucrose gradient. The samples were analyzed by SDS-PAGE and immunoblotting (anti-PDR5 antibody).
(TIF)

S10 Fig Sucrose gradient fractions 16 to 27 of PDR5 containing *S*. *cerevisiae* crude membranes. Crude membranes were separated via ultracentrifugation through a multistep sucrose gradient. The samples were analyzed by SDS-PAGE and immunoblotting (anti-PDR5 antibody).
(TIF)
