# Supplementary figures and images for: Cloning and expression of selected ABC transporters from the Arabidopsis thaliana ABCG family in Pichia pastoris
Source: PLoS One. 2019 Jan 18;14(1):e0211156. doi: 10.1371/journal.pone.0211156 (PMC6338384; doi:10.1371/journal.pone.0211156)

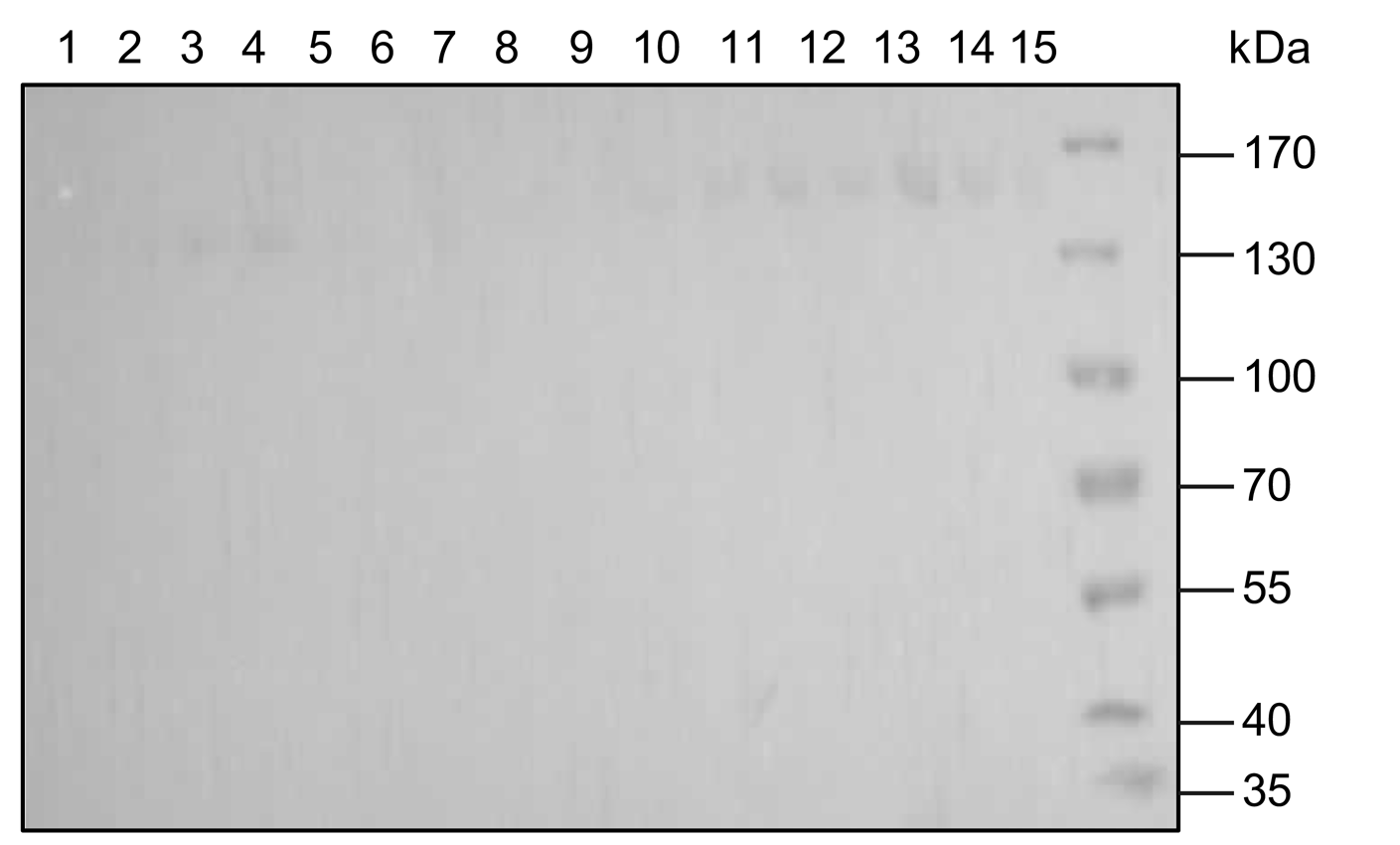

Supplement: S1 Fig — Crude membranes were separated via ultracentrifugation through a multistep sucrose gradient. The samples were analyzed by SDS-PAGE and immunoblotting (anti-His-tag antibody). (TIF) [file pone.0211156.s001.tif]

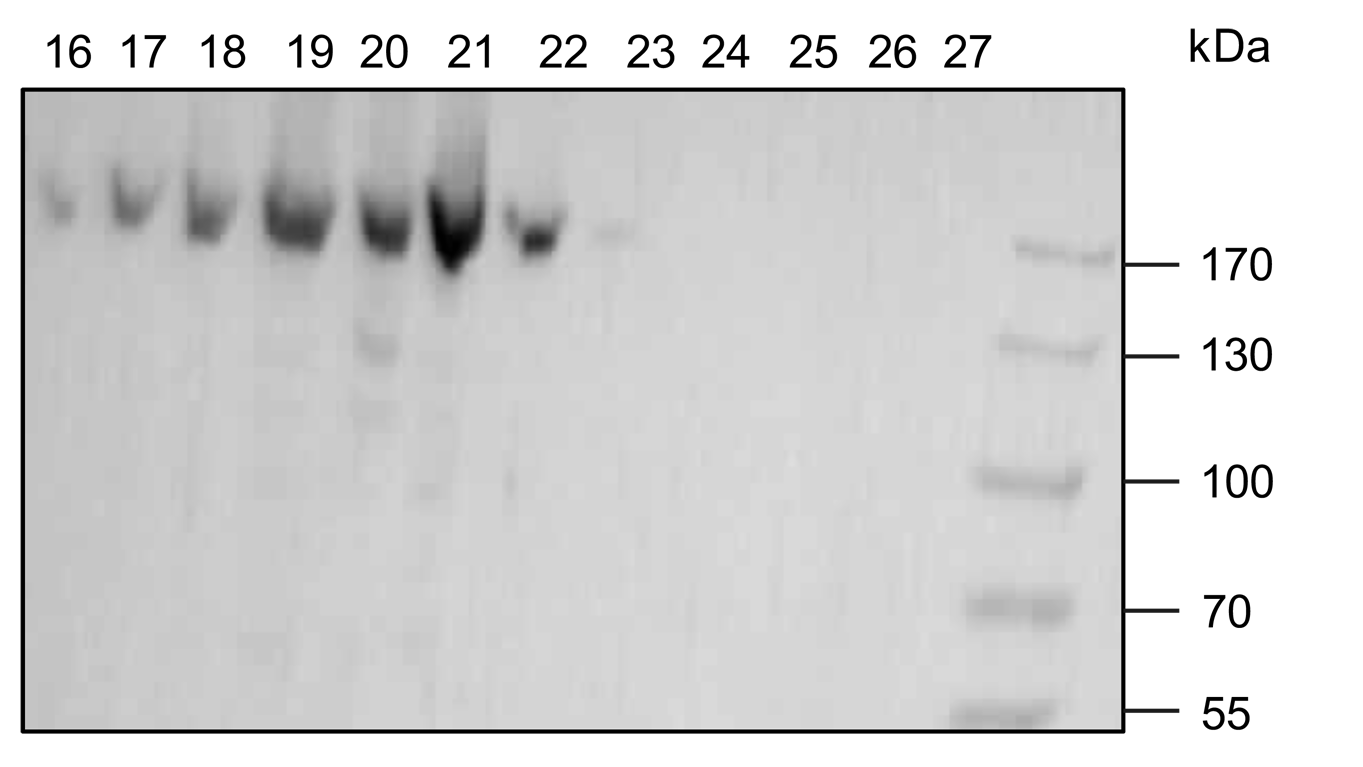

Supplement: S2 Fig — Crude membranes were separated via ultracentrifugation through a multistep sucrose gradient. The samples were analyzed by SDS-PAGE and immunoblotting (anti-His-tag antibody). (TIF) [file pone.0211156.s002.tif]

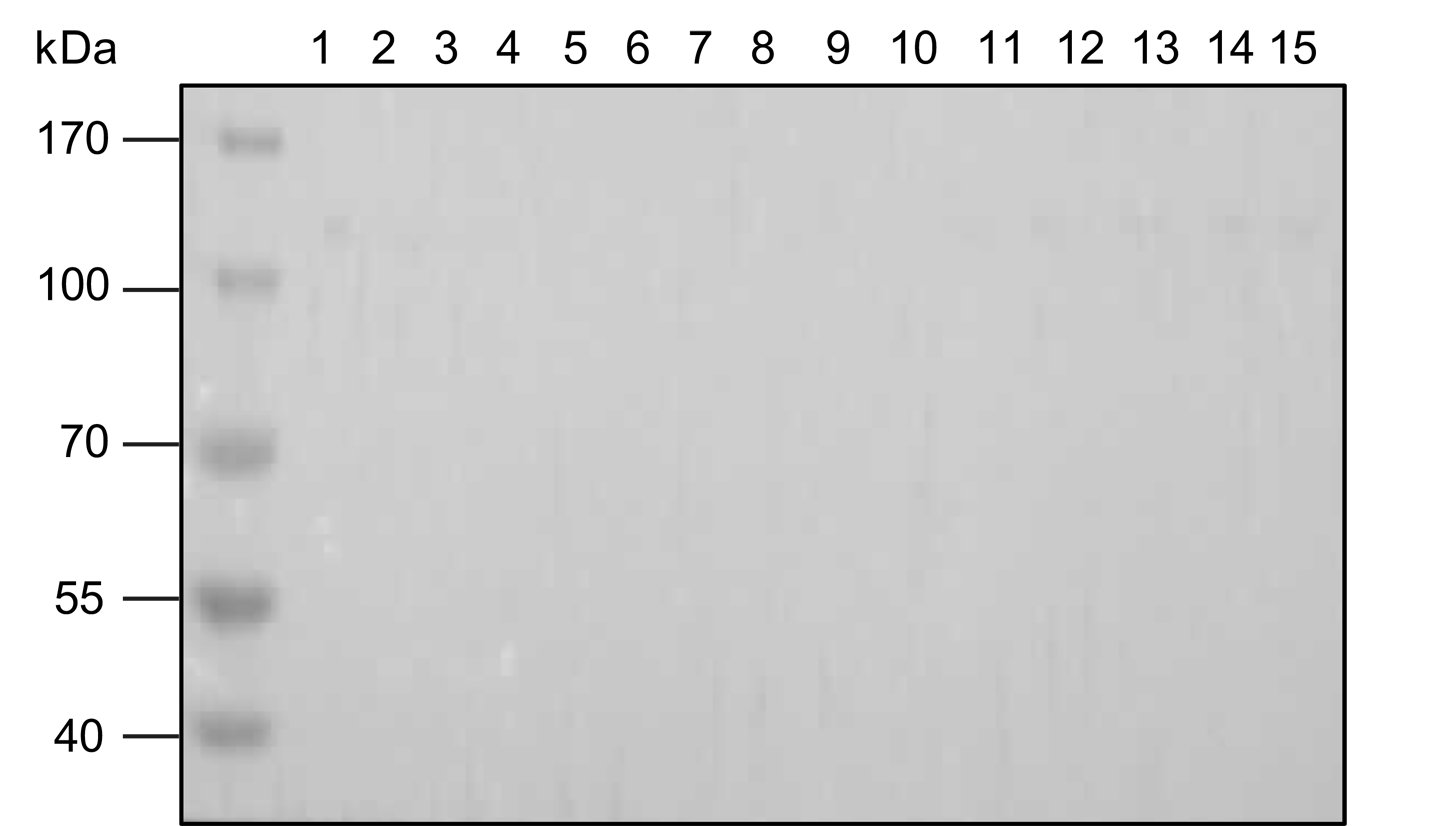

Supplement: S3 Fig — Crude membranes were separated via ultracentrifugation through a multistep sucrose gradient. The samples were analyzed by SDS-PAGE and immunoblotting (anti-His-tag antibody). (TIF) [file pone.0211156.s003.tif]

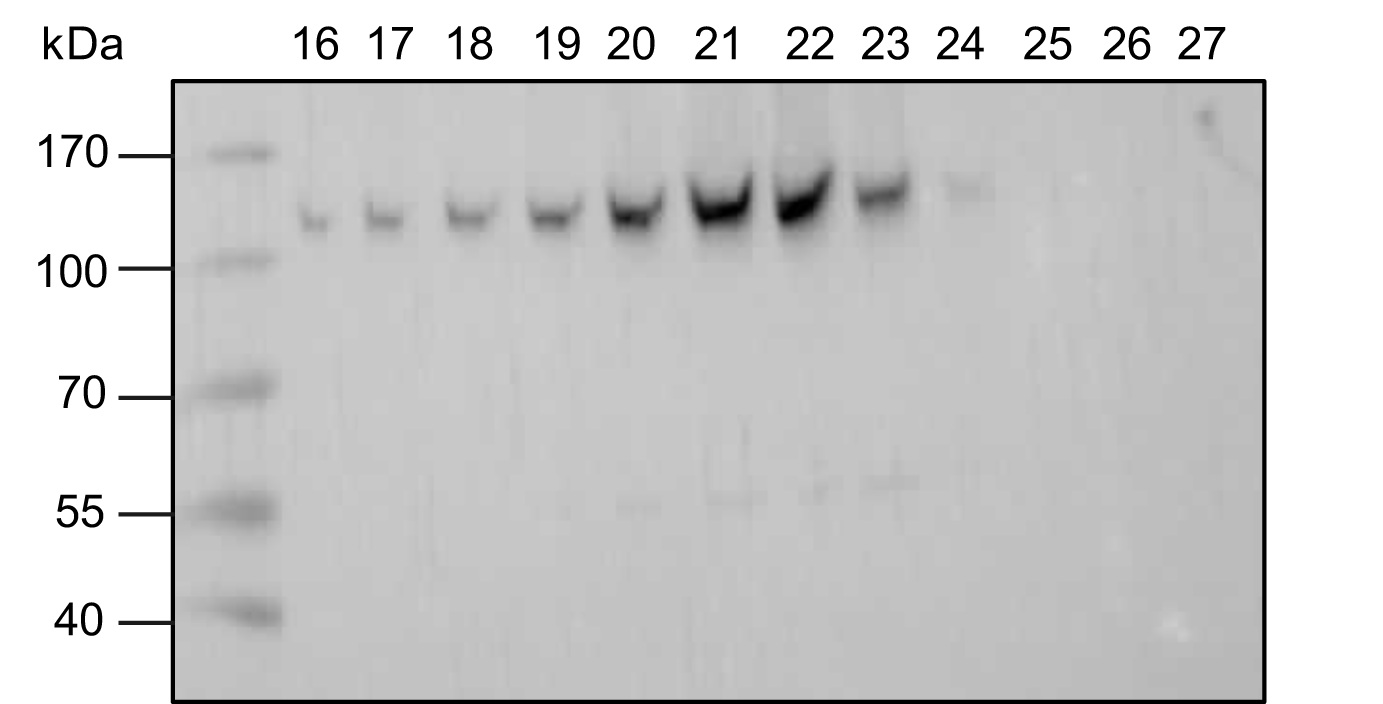

Supplement: S4 Fig — Crude membranes were separated via ultracentrifugation through a multistep sucrose gradient. The samples were analyzed by SDS-PAGE and immunoblotting (anti-His-tag antibody). (TIF) [file pone.0211156.s004.tif]

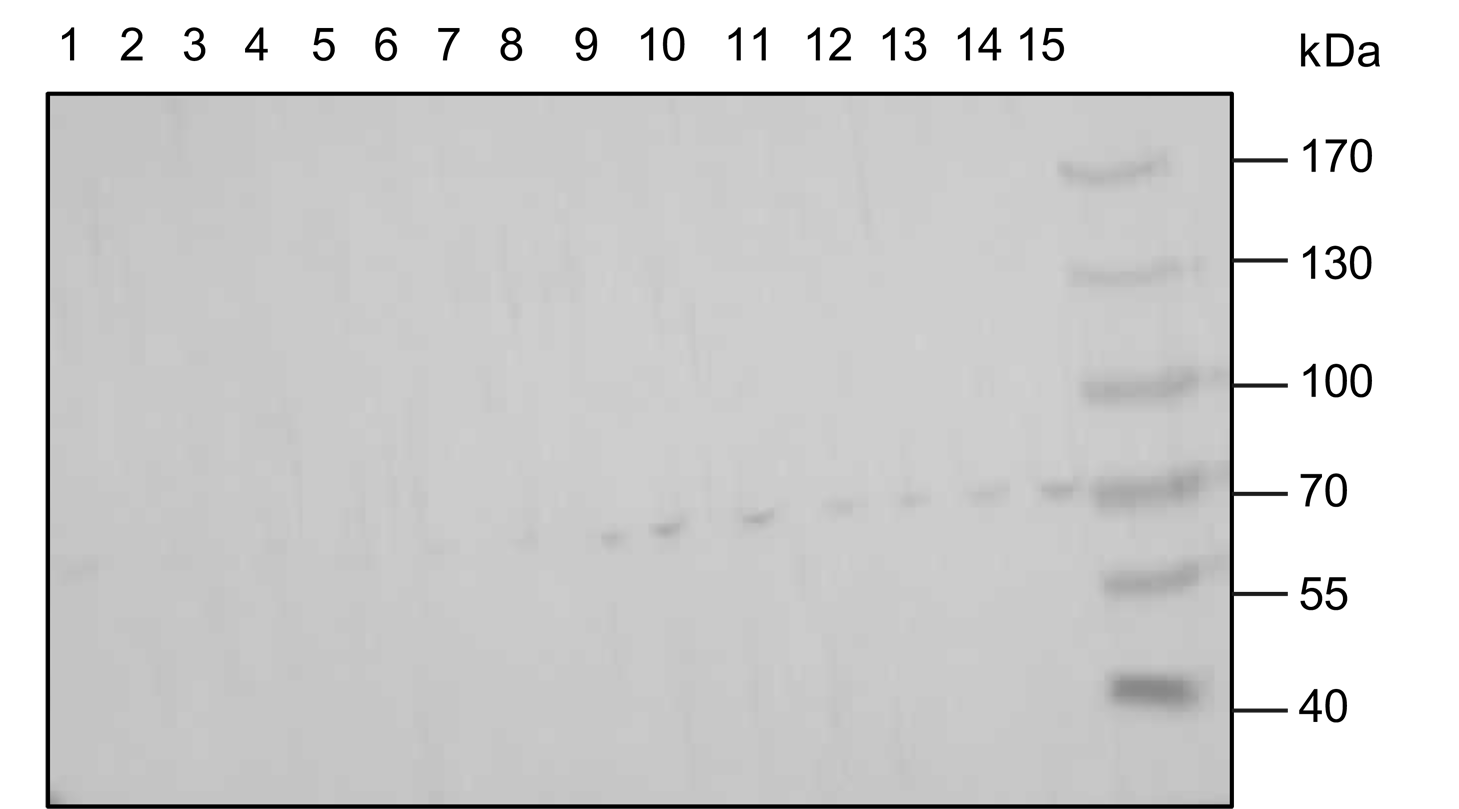

Supplement: S5 Fig — Crude membranes were separated via ultracentrifugation through a multistep sucrose gradient. The samples were analyzed by SDS-PAGE and immunoblotting (anti-His-tag antibody). (TIF) [file pone.0211156.s005.tif]

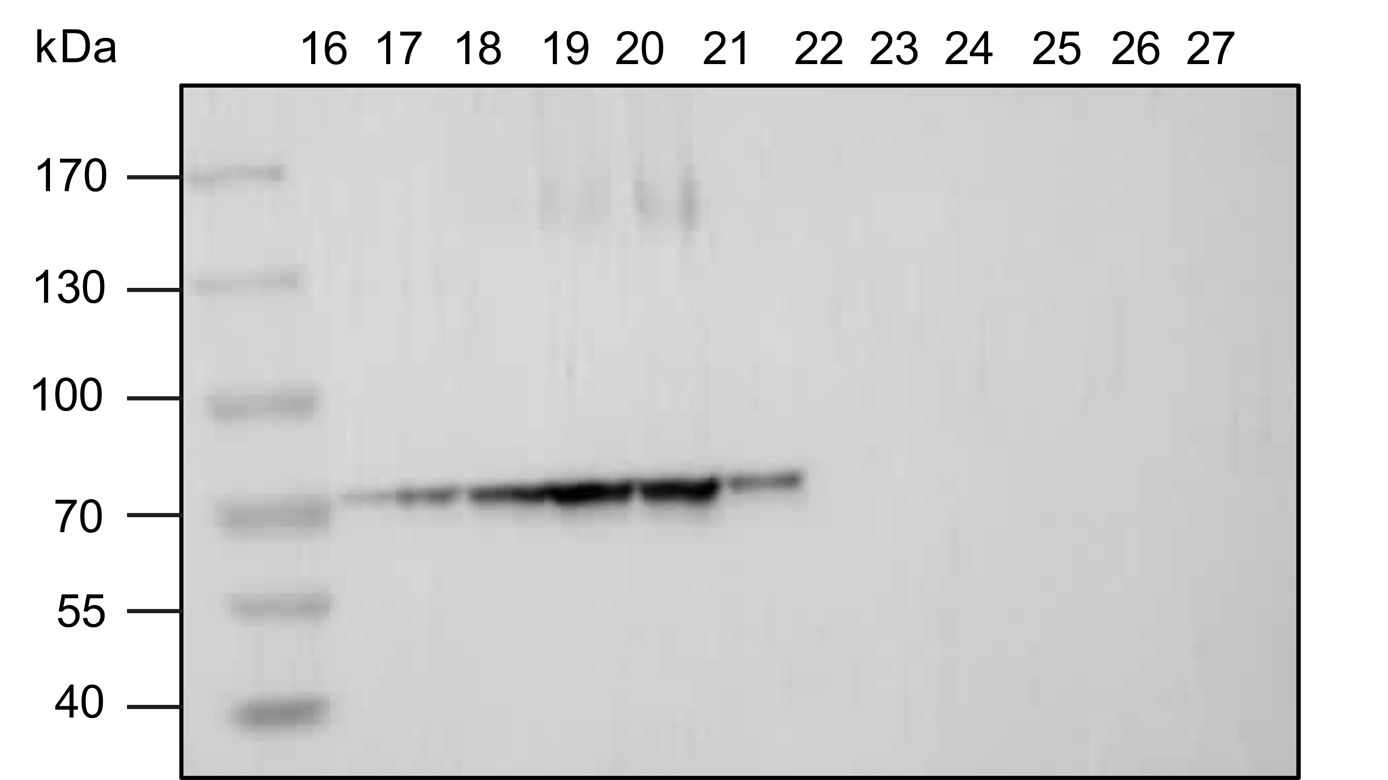

Supplement: S6 Fig — Crude membranes were separated via ultracentrifugation through a multistep sucrose gradient. The samples were analyzed by SDS-PAGE and immunoblotting (anti-His-tag antibody). (TIF) [file pone.0211156.s006.tif]

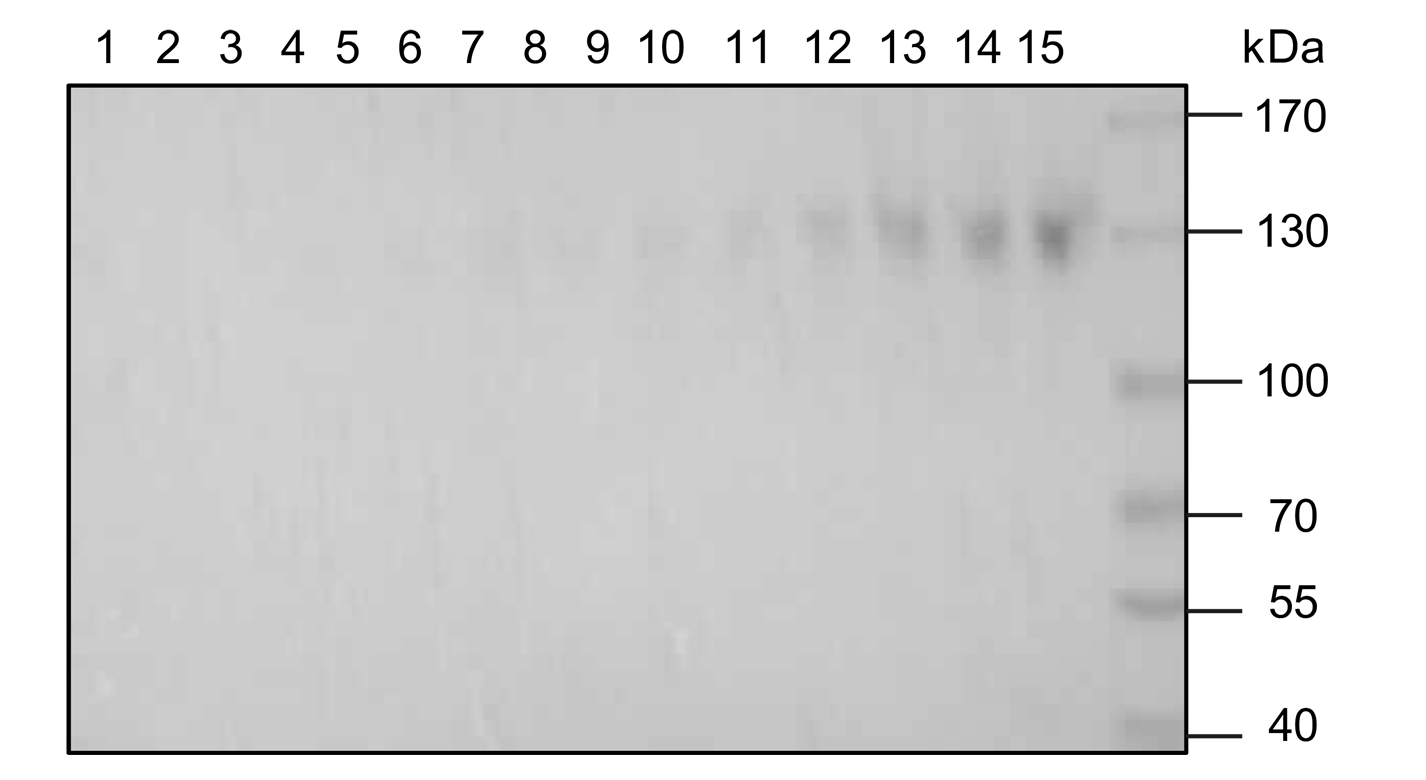

Supplement: S7 Fig — Crude membranes were separated via ultracentrifugation through a multistep sucrose gradient. The samples were analyzed by SDS-PAGE and immunoblotting (C219 antibody). (TIF) [file pone.0211156.s007.tif]

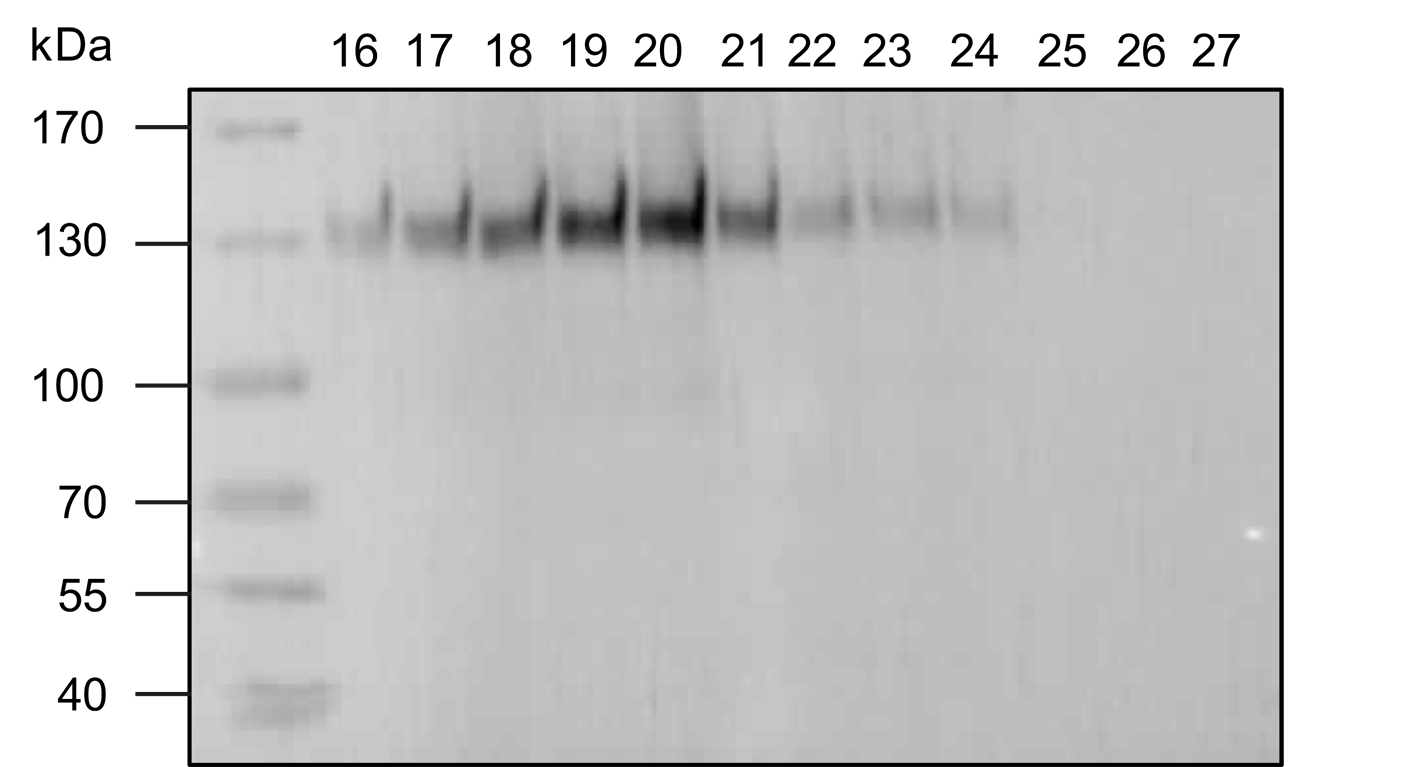

Supplement: S8 Fig — Crude membranes were separated via ultracentrifugation through a multistep sucrose gradient. The samples were analyzed by SDS-PAGE and immunoblotting (C219 antibody). (TIF) [file pone.0211156.s008.tif]

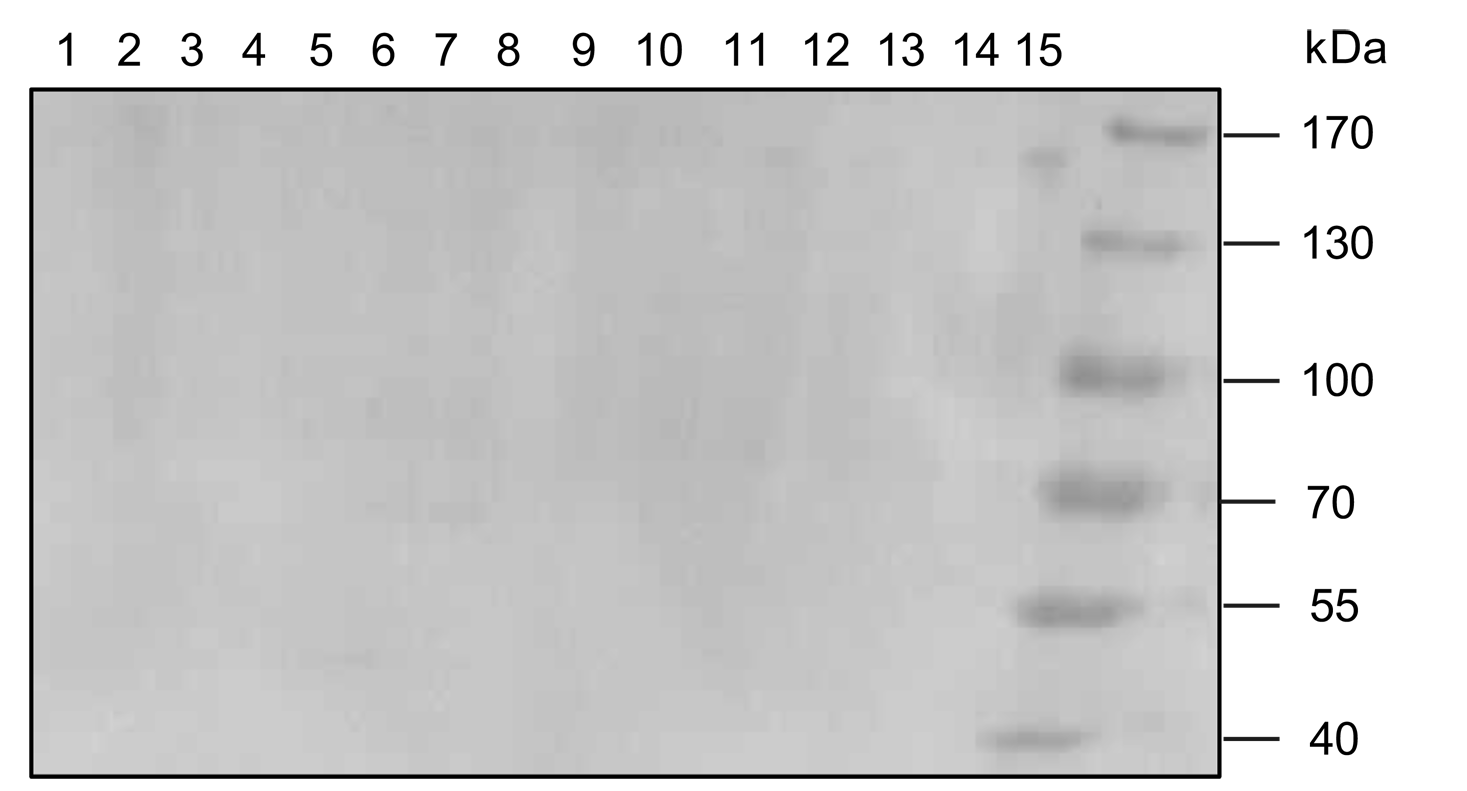

Supplement: S9 Fig — Crude membranes were separated via ultracentrifugation through a multistep sucrose gradient. The samples were analyzed by SDS-PAGE and immunoblotting (anti-PDR5 antibody). (TIF) [file pone.0211156.s009.tif]

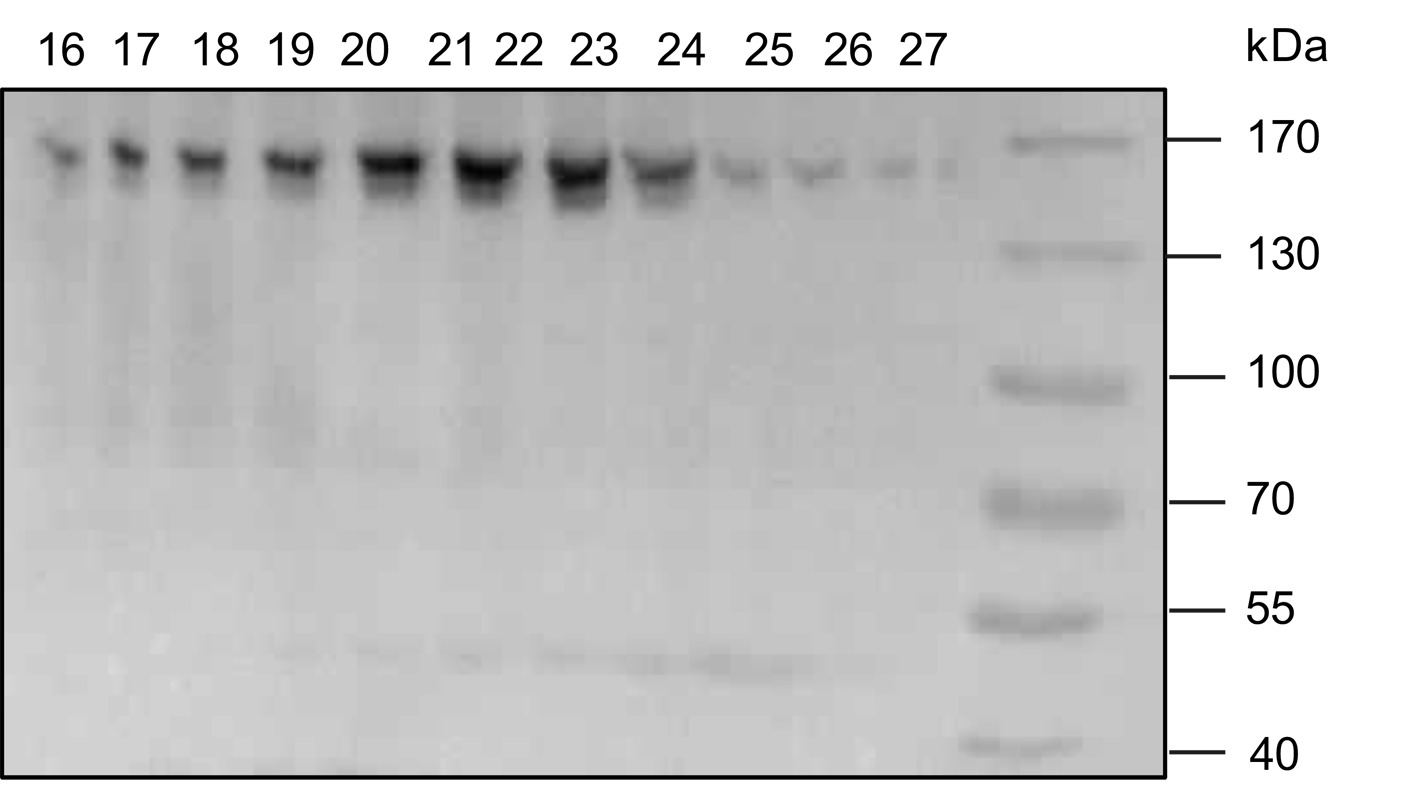

Supplement: S10 Fig — Crude membranes were separated via ultracentrifugation through a multistep sucrose gradient. The samples were analyzed by SDS-PAGE and immunoblotting (anti-PDR5 antibody). (TIF) [file pone.0211156.s010.tif]
